# Latent neural population dynamics underlying breathing, opioid-induced respiratory depression and gasping

**DOI:** 10.1038/s41593-023-01520-3

**Published:** 2024-01-05

**Authors:** Nicholas Edward Bush, Jan-Marino Ramirez

**Affiliations:** 1 Center for Integrative Brain Research, Seattle Children’s Research Institute, Seattle, WA USA; 2 Department of Pediatrics, University of Washington, Seattle, WA USA; 3 Department of Neurological Surgery, University of Washington, Seattle, WA USA

**Keywords:** Central pattern generators, Respiration, Neurophysiology, Neural circuits, Dynamical systems

## Abstract

Breathing is vital and must be concurrently robust and flexible. This rhythmic behavior is generated and maintained within a rostrocaudally aligned set of medullary nuclei called the ventral respiratory column (VRC). The rhythmic properties of individual VRC nuclei are well known, yet technical challenges have limited the interrogation of the entire VRC population simultaneously. Here we characterize over 15,000 medullary units using high-density electrophysiology, opto-tagging and histological reconstruction. Population dynamics analysis reveals consistent rotational trajectories through a low-dimensional neural manifold. These rotations are robust and maintained even during opioid-induced respiratory depression. During severe hypoxia-induced gasping, the low-dimensional dynamics of the VRC reconfigure from rotational to all-or-none, ballistic efforts. Thus, latent dynamics provide a unifying lens onto the activities of large, heterogeneous populations of neurons involved in the simple, yet vital, behavior of breathing, and well describe how these populations respond to a variety of perturbations.

## Abstract (web summary)

Bush and Ramirez show that the activity of neuronal populations implicated in generating breathing evolves along a rotational low-dimensional trajectory that is stably maintained even when challenged with opioids but collapses during gasping.

## Main

Recent advances in the ability to record the activity of large populations of neurons have driven concurrent adoption of dimensionality reduction techniques to describe complex population dynamics^1–4^. These approaches have been widely successful across many systems to uncover simple attractor-like dynamics that describe neural population activity.

Additionally, studies from small invertebrate neuronal networks have demonstrated that qualitatively and quantitatively similar patterns of rhythmic activity can be observed over a large regime of varying combinations of biophysical properties, suggesting that neuronal networks maintain specific patterns of activity that can emerge from many different sets of synaptic strengths and intrinsic membrane properties^5^. That is, numerous solutions for a given neural behavior can arise from a degenerate space of constituent cellular components.

The neural circuits that underly breathing in mammals afford a tractable handle on the intersection of these two complementary descriptions of redundancy underlying neural processes. Breathing is a rhythmic, stereotyped motor behavior that is constitutively maintained throughout the life of an animal. Despite the relative simplicity of this behavior, the neural circuits that drive and maintain breathing are distributed throughout anatomically distributed and molecularly diverse neural populations that compose the rostrocaudally extended ventral respiratory column (VRC)^6–10^. The VRC contains vital centers that act as the scaffold for maintaining breathing, including the intrinsically rhythmic, bilaterally synchronized, preBötzinger complex (preBötC)^11–13^, the chemo-sensitive retrotrapezoid nucleus (also termed the parafacial respiratory group)^14^ and bulbospinal premotor neurons of the ventral respiratory group^15^. The VRC also participates in critical bidirectional integration with medullary, pontine, thalamic and cortical centers^16–18^, as well as the peripheral nervous system^19,20^. This vast interconnectivity imbues the respiratory centers with sufficient flexibility to support varied control of breathing during behaviors such as whisking and vocalizing^21,22^.

In this Article, we introduce a novel experimental preparation that allows for large-scale electrophysiological recordings along the rostrocaudal extent of the medulla. We combine recordings from Neuropixel probes^23^ with optogenetic tagging^24^ and 3D histological reconstructions (Methods) to detail the respiratory-related activities of identified VRC neural populations in freely breathing mice with intact trachea and vagal nerves. Our results show that VRC population activity evolves along a continuous, rotational trajectory on a low-dimensional neural manifold that is consistent across animals and recordings. Inspiratory and expiratory activity can be described by intersecting, linear dynamical systems governed by rotational dynamics. Notably, the discrete phase transition into expiration forms an attractive target in neural population space. We thus expand on the classical idea of the ‘inspiratory off-switch’^25–27^ such that the offset of inspiration is the critical temporal reorganizing feature for dynamics of the entire VRC population. The rotational trajectories, however, reflect a continuous and gradual transition of neuronal population activity from the expiratory through the inspiratory phase.

This approach provides novel insights into the coordinated activity of respiratory neural populations and allows us further to test how these rotational dynamics are disrupted by systemic, physiologically relevant perturbations to respiratory dynamics: opioids and hypoxia. Opioid-induced respiratory depression (OIRD) is a leading cause of death in the United States^28,29^ and our understanding of the molecular, cellular and circuit mechanisms of OIRD is continually evolving^30–33^. Here we show that opioids cause complex, diverse and intractable changes in the firing activity of single units. However, when viewed at the level of the low-dimensional population dynamics, the trajectories are preserved, but slowed, suggesting that there are redundant network compensations to preserve respiratory function in response to perturbations.

In contrast, acute hypoxia induces dramatic physiological pressure that alters the normal breathing pattern to produce auto-resuscitative gasping^34,35^. The failure to rouse during gasping is thought to underlie susceptibility to sudden unexpected infant death syndrome (SUIDS)^36–38^. Our data show that, during gasping, the rotational dynamics observed during eupnea (normal breathing) collapse towards the inspiratory-off attractor and gasps manifest as ballistic, all-or-nothing, monophasic excursions in the latent state.

Together, these data provide a novel and comprehensive survey of the constituent neural populations in the ventrolateral medulla that are involved in breathing and place the neural circuits that govern breathing in the context of the burgeoning field of neural population dynamics.

## Mapping of anatomically and optogenetically identified VRC units

**Fig. 1:**
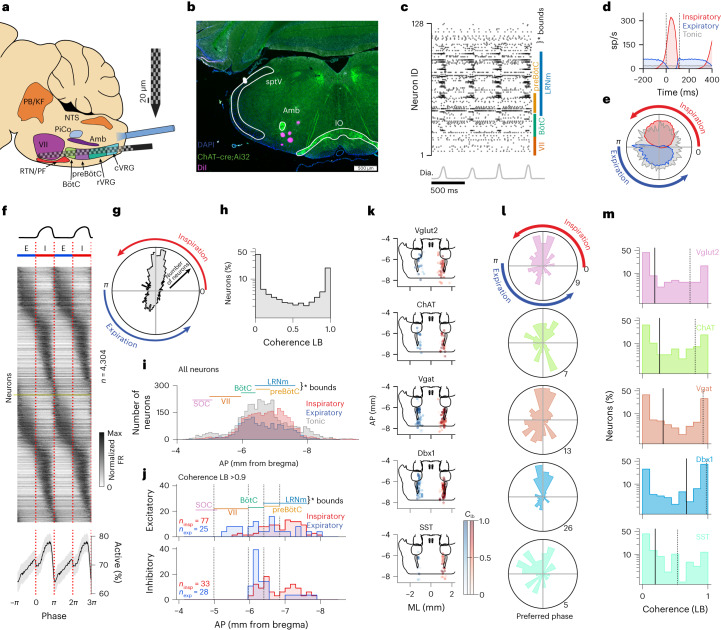
Large-scale recording to characterize VRC populations. **a**, Recording schematic. **b**, Coronal section showing recording tracks (purple) from 5 sequential VRC recordings. **c**, Raster of 128 units with corresponding integrated diaphragm EMG (gray). Raster is ordered rostral (bottom) to caudal (top) and approximate rostral–caudal boundaries of landmark regions are shown. **d**, Diaphragm onset aligned histogram for three units (red: inspiratory, blue: expiratory, gray: tonic). Mean firing rate (FR) ± s.e.m. s.e.m. is not visible. sp/s, spikes/second. **e**, Phasic tuning curves of the units in **d**. Radial axis is maximum normalized spike rate. Angular axis is breath phase. **f**, Phase-aligned, maximum normalized responses of phasic (*C*_lb_ > 0.1), somatic units across all recordings (*n* = 4,304). Responses are duplicated in both axes to visualize phasic boundaries. Bottom shows percent of neurons active at a given phase, where active is defined as >10% maximum firing rate. Shaded region is mean ± 100× s.e.m. **g**, Angular histogram of preferred phase of all phasic somatic units. **h**, Distribution of the *C*_lb_ for all somatic units (*n* = 8,001). **i**, Marginal distributions of all inspiratory (red), expiratory (blue) and tonic (gray) somatic units recorded along the AP axis (100 μm bins). AP boundaries of landmark regions are shown, but histograms include units from all regions that overlap at a given AP location. For example, LRNm and preBötC overlap. LRNm, lateral reticular nucleus, medial division. **j**, AP distributions of strongly coherent somatic units (*C*_lb_ > 0.9) identified by opto-tagging as excitatory (Vglut2^+^ or Dbx1^+^, top) or inhibitory (Vgat^+^, bottom) (red: inspiratory; blue: expiratory). **k**, Location and respiratory coherence of positively tagged phasic somatic units. Expiratory units (blue) are shown on the left, inspiratory (red) units on the right for visualization; all units were recorded from the left medulla. Dot saturation indicates the coherence of that unit (color bars are *C*_lb_). **l**, Angular histograms of preferred phase for all phasic, positively tagged somatic units. Angular axis is preferred phase, and radial axis is number of units. **m**, Distributions of *C*_lb_ for all positively tagged somatic units. Black vertical line is median; dashed vertical line is 75th percentile. Source data

Neuropixel probes^23^ were inserted along the rostrocaudal axis of the VRC of 36 urethane anesthetized mice to record single unit activity from the entire VRC simultaneously (Fig. 1a, Supplementary Fig. 1 and Supplementary Video 1). Urethane anesthesia sustains respiratory rates close to those observed in awake animals^39^ (approximately 2–4 Hz) and maintains many cardiorespiratory reflexes. Breathing was monitored with diaphragm electromyogram (EMG). Across all animals, we analyzed 15,299 single units in 139 separate recordings (average 3.8 recordings per animal, 114 units per recording). Repeated insertion of the Neuropixel probe did not severely alter respiratory activity (Supplementary Fig. 1). We identify units as putatively axonal (*n* = 7,298) or somatic (*n* = 8,001) in origin on the basis of their extracellular waveform shape^40^ by modifying a method recently described by Sibille et al.^41^ (Supplementary Fig. 2). Further analyses in which somatic and axonal units are combined will refer generally to ‘neurons’ or ‘units’. Fluorescent DiI was used to identify the locations of each probe insertion in post-hoc histological analyses (Fig. 1b and Supplementary Fig. 1). In addition, we use opto-tagging^24^ to identify a subset of recorded neurons by their transcriptional markers (Supplementary Fig. 2h–j). We identified 192/2,504 (7.7%) Vglut2^+^; 156/2,866 (5.4%) ChAT^+^; 334/5,774 (5.8%) Vgat^+^; 346/2,192 (15.8%) Dbx1^+^; and 106/2,469 (4.3%) SST^+^ units. Dbx1^+^ and SST^+^ units are of interest as they are involved in generating breathing rhythm and pattern, respectively^15,42–44^.

An example raster of 128 simultaneously recorded single units is shown in Fig. 1c, along with integrated diaphragm for four breaths. As expected, single units exhibit varied breath-averaged activity patterns (Fig. 1d). Since respiratory rate and inspiratory durations vary over time and across animals, we map each breath to phase (*ϕ*) such that the onset of inspiration occurs at *ϕ* = 0, inspiratory bursts have 0 ≤ *ϕ* ≤ *π* and inter-breath intervals (IBIs) (referred to as expiration throughout) have −*π* < *ϕ* < 0. We avoid more granular discretization of phase (for example, pre-inspiratory, which has no quantitatively defined temporal marker) in favor of reporting the exact *ϕ* values. We then quantify each neuron’s activity as a function of this normalized phase (Fig. 1e–h). Individual neurons are parameterized by coherence (quantifies how strongly a neuron shares frequency components with breathing; Supplementary Fig. 3), and phase lag (that is, phase of breathing cycle in which the neuron’s activity is biased). We compute the coherence and phase lag with Chronux (Methods) and use the lower bound on coherence (*C*_lb_; 99.9% confidence interval) so as to not categorize some neurons as more coherent than statistically likely. Neurons with *C*_lb_ > 0.1 were categorized to be phasic, and any neuron with *C*_lb_ ≤ 0.1 to be tonic; this categorization is used throughout. We choose a low value of *C*_lb_ to include neurons that may be only weakly correlated with breathing (Supplementary Fig. 3b). Putative axonal units had lower respiratory coherence and firing rates than putative somatic units (Supplementary Fig. 2e,f). Slightly more than half of all somatic units showed at least minor evidence of respiratory coherence (4,304 of 8,001, 53%). Phasic somatic neurons were more likely to be inspiratory (2,586 of 4,304; 60%) (Fig. 1g,h). On average, inspiratory units have higher firing rates and are more coherent than expiratory units (Supplementary Fig. 3c,d,g).

The phase-averaged and maximum rate normalized activities of all putatively somatic, coherent (*C*_l__b_> 0.1) units smoothly tile the respiratory cycle (Fig. 1f). Notably, there is sequential recruitment of neurons during progression of the inspiratory (I) phase. While the inspiratory and expiratory phases recruit largely nonoverlapping neural populations, the boundary from expiration to inspiration is straddled by many neurons. By contrast, fewer neurons straddle the inspiration-to-expiration transition (Fig. 1f). Thus, the onset of inspiration is a gradual, continuous neural process while the offset of inspiration is a sharp transition.

**Extended Data Fig. 1:**
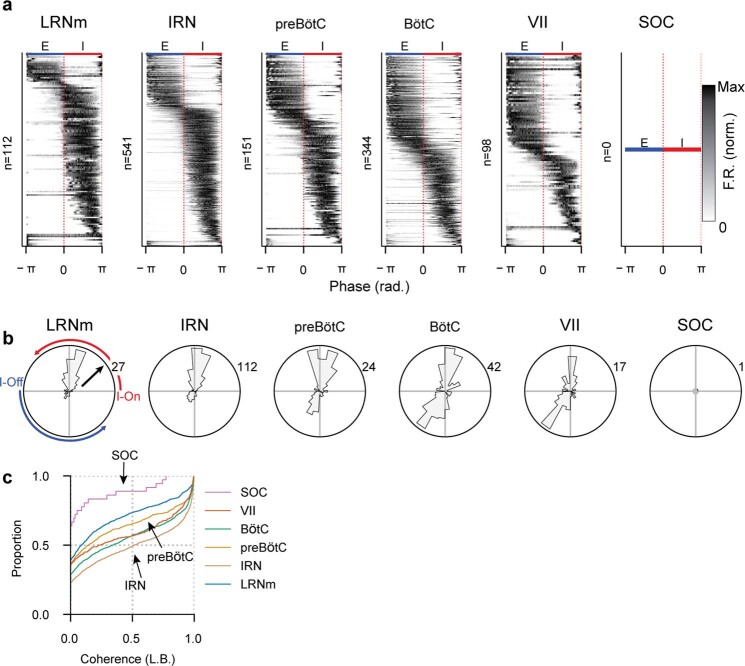
Phasic activity patterns of strongly coherent somatic units parcellated by VRC sub-regions. (**a**) Phase-averaged, maximum normalized activity of all strongly coherent (C_lb_>0.9) somatic units found in each VRC subregion. As expected, no superior olivary complex (SOC) neurons are strongly coherent. Regions are arranged from caudal (left) to rostral (right). Proportionally more units are expiratory in more rostral regions. (**b**) Angular distribution of units with a given phase lag (*ϕ*) with respect to breathing, for all regions in (a). Angular axis is respiratory phase, radial axis is number of units with a given *ϕ* value. Inspiration is top half of circle (*ϕ* > 0), expiration is bottom half of circle (*ϕ* < 0) (**c**) Cumulative distributions of the C_lb_ values for all somatic units in each region. Rightward shifted curves indicate a higher proportion of coherent units.

**Extended Data Fig. 2:**
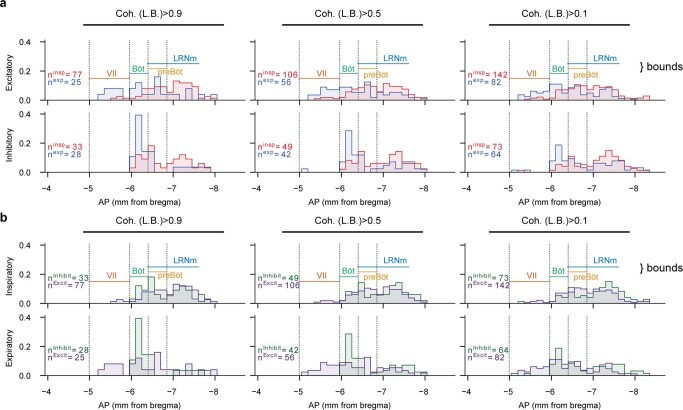
Anterior-posterior distributions of somatic units of identified genotype and respiratory activity pattern. Given that Dbx1^+^ neurons are glutamatergic, we combine Dbx1^+^ opto-tagged neurons with Vglut2^+^ opto-tagged neurons to identify the excitatory units. (**a**) AP distributions of inspiratory (red) and expiratory (blue) units that are excitatory (top) or inhibitory (bottom) for varying coherence thresholds (left to right). Y-axis is probability of a unit being in a given bin (150 *μm*), normalized within the identified group (that is, inspiratory or expiratory are normalized independently). Of note is the high proportion of inhibitory neurons in the canonical Bötzinger complex, particularly if analyses are restricted only to strongly coherent neurons, and the high proportion of inspiratory neurons caudal to the preBötC, likely including bulbospinal rVRG premotor populations. AP extent of landmark regions in the ventral medulla are shown, but histograms include units from all regions that overlap at a given AP location (**b**) Same data as in (**a**), but colored by excitatory (purple)/inhibitory (green); inspiratory neurons in top row, expiratory neurons in bottom.

**Extended Data Fig. 3:**
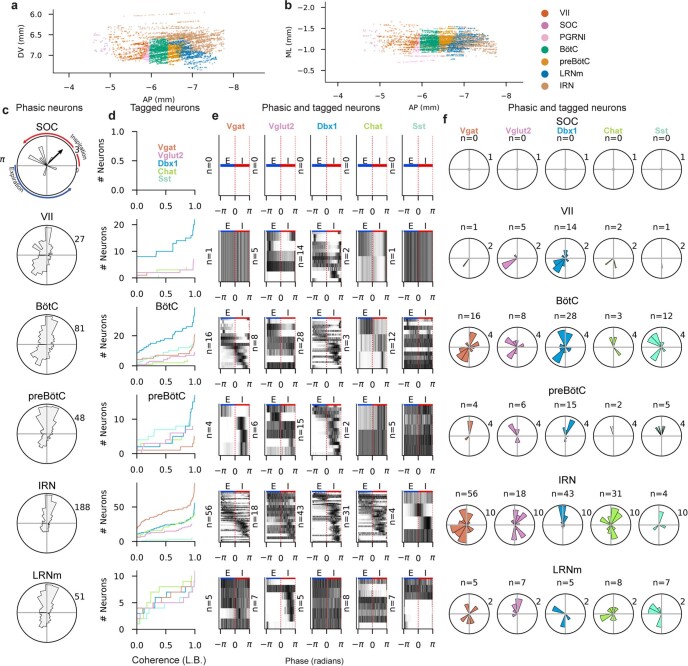
Detailed functional attributes of anatomically and optogenetically identified, putatively somatic units. (**a,b**) As the preBötC and BötC are not defined in the AllenCCF, we subdivide the Paragigantocellular Reticuclar Nucleus (lateral) (PGRNl) region into preBötC and BötC (See Methods) (**a**) Saggital and (**b**) horizontal projection of all recorded units colored by their identified subregion. (**c**) Distribution of preferred phase of all phasic somatic units (*C*_*lb*_ > 0.1) in the given region. Proportionally more preBötC neurons are inspiratory. Radial axis is number of neurons; radial label is maximal units per bin; angular axis is preferred phase. (**d**) Cumulative counts of lower bound of coherence values for all opto-tagged somatic units in a given region, colored by their genotype. Y-axis is number of units. Right shifted curves represent a greater proportion of the population are strongly coherent (for example, Vgat^+^ BötC units are strongly coherent as a population). (**e**) Phasic activity patterns of all phasic (*C*_*lb*_ > 0.1) opto-tagged units in each region. Each panel is ordered by preferred phase. (**f**) Polar histograms of preferred phase for all phasic, opto-tagged units in (e); angular axis is preferred phase, radial axis is number of neurons and radial label is maximal number of units per bin as in (c).

We next determined the anatomical location of each single unit. We reconstruct the 3D location of the fluorescent probe tract with respect to the AllenCCF and identify the location of the channel on which the neuron’s spike waveform was largest. Inspiratory and expiratory neurons were found continuously distributed throughout the anterior–posterior (AP) axis of the VRC (Fig. 1i, Supplementary Fig. 3 and Extended Data Figs. 1–3). As expected, proportionally more phasic neurons were found along the anatomical bounds of the VRC, while tonic neurons were more widely spatially distributed (Fig. 1i, Supplementary Fig. 3, Extended Data Figs. 1–3 and Supplementary Video 2). These distributions were not qualitatively affected by varying the coherence threshold (Supplementary Fig. 3a). However, as the coherence threshold is raised (that is, we consider only strongly coherent neurons in the analysis), classical parcellations of the VRC become more pronounced (Supplementary Fig. 3 and Extended Data Fig. 2). In all locations, inspiratory neurons were more numerous than expiratory neurons. There is a predominant rostrocaudal gradient where expiratory neurons are found rostrally, while inspiratory neurons are found caudally (Supplementary Fig. 3 and Extended Data Figs. 1 and 2).

Lastly, we assessed the respiratory related neural activity patterns and anatomical distribution of optogenetically identified, putatively somatic units. In general, units of all optogenetic identities and phasic firing patterns are found widely distributed throughout the VRC (Fig. 1j–l). However, some populations overrepresent certain phases of breathing (Fig. 1l and Extended Data Fig. 3e,f). Of particular note is the small population of SST^+^ expiratory neurons that were found primarily just caudal to the facial nucleus (VII), while inspiratory SST^+^ neurons were found throughout the AP axis. On average, Vgat^+^ and Dbx1^+^ neurons are more strongly respiratory tuned than the Vglut2^+^, ChAT^+^ or untagged populations (Fig. 1m). Vgat^+^ neurons overrepresent expiratory phase; and inhibitory, expiratory neurons tend to be—but are not exclusively—found in the rostral VRC (that is, Bötzinger complex (BötC)/parafacial respiratory group). Dbx1^+^ neurons overrepresent the inspiratory phase, and these inspiratory neurons are found more caudally in the VRC.

## Activity-based clustering reveals continuously distributed activity patterns

**Fig. 2:**
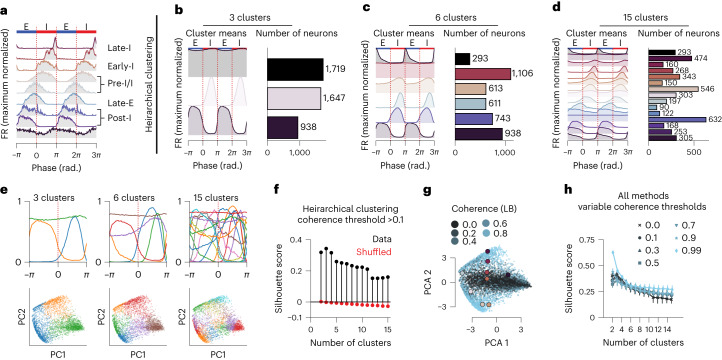
Clustering of phasic units reveals continuum of activity patterns. All data are for somatic units only. **a**, Nine example phasic (coherence lower bound >0.1) units with classical activity patterns with respect to breathing phase. Firing rates (FR) are maximum normalized. Classical activity pattern identities are given on the right on the basis of manual identification. **b**–**d**, Hierarchical clustering of phasic activity patterns showing the mean activity pattern for a given cluster (left), and the number of units assigned to that cluster for clustering into 3 (**b**), 6 (**c**) and 15 (**d**) clusters (right). Separating units into three clusters results in inspiratory, expiratory and tonic neurons. Six clusters result in clusters that resemble classical post-inspiratory, tonic, pre-inspiratory, inspiratory and expiratory, while 15 clusters result in a continuum of clusters that tile the breathing phase with both incrementing and decrementing patterns^6,7,25,46^ with varying phasic structure. **e**, Cluster means (top) and projection (bottom) of phasic tuning curves into two-dimensional PC space, colored by cluster identity as in top. Each dot is the PCA projection of the phase-averaged activity of a single unit. Colors indicate assigned clusters in the exemplar cases for 3, 6 and 15 clusters. (NB: clustering here is performed on the raw phase average responses, and the PC projections are for visualization.) **f**, Maximal silhouette score for hierarchical clustering for 2 through 15 computed clusters. The maximal silhouette score occurs when the number of clusters is 3. Red points indicate shuffled control. **g**, PCA projections of the phasic activity patterns for all units, colored by lower bound (LB) of respiratory coherence. Highlighted points in **g** correspond to the example units of the corresponding color in **a**. **h**, Clustering is repeated with multiple clustering and data processing techniques (*n* = 6 techniques, error bars are 95% confidence interval), for multiple coherence threshold values. The silhouette scores for those techniques are shown as a function of number of clusters. When weakly coherent units are included (coherence threshold 0.1, 0.3, 0.5), the optimal number of clusters is 3 (inspiratory, expiratory and tonic). However, if only strongly coherent units are included (coherence threshold [0.7, 0.9, 0.99]), the optimal number of clusters is 2 (inspiratory and expiratory), as the tonic neurons have been excluded before clustering. Source data

VRC units are typically categorized into one of several groups based on their phase-averaged activity pattern. These categories describe the relationship between the unit’s activity and phase and allow for comparisons across units. For example, a typical pre-inspiratory unit will exhibit a ramp in firing rate before the onset of the breath (or burst in vitro) and decay during the inspiratory effort (Fig. 2a). While many studies agree qualitatively on inclusion of units into these categories^7,45,46^, the number of categories varies across studies, and no quantitative categorization scheme exists. Several units that exemplify some of these categories are shown in Fig. 2a.

The ability to record from thousands of respiratory neurons affords data-driven clustering methods for identifying groups of neurons that exhibit similar phase-aligned activity. We first normalize the binned activity (100 bins, phase-averaged activity from −*π* to *π*) of each somatic unit between zero and one. We perform both hierarchical clustering and *k*-means clustering on both the full data (each unit has 100 features, one for each phase bin) and two sets of reduced data (Methods). We vary the number of discovered clusters from 2 to 15 to determine the optimal number of activity-based clusters as defined by maximal silhouette score.

To exemplify the results of such a clustering approach, we find that splitting into three clusters returns inspiratory, expiratory and weakly phasic cluster means; splitting into 6 clusters returns cluster means consistent with classical categories; and splitting into 15 clusters returns cluster means that sequentially tile phase (Fig. 2b–e). We compute the silhouette score and find that the optimal number of clusters is 3 (Fig. 2f,h), which is fewer than is typically described and clearly inadequate to account for the heterogeneity seen in the phase-averaged activities. However, further discretization oversplits the data. In this analysis we include even weakly coherent units that could contaminate the clustering and give an erroneous appearance of continuity in the population (Fig. 2g). Therefore, we systematically vary a coherence threshold such that we cluster only on increasingly exclusive populations of increasingly coherent units. We find that as weakly coherent units are excluded, the maximal silhouette score becomes two, and the cluster means resemble inspiratory and expiratory units only (Fig. 2h). While two clusters of coherent neurons are quantitatively supported, such a dichotomy clearly underrepresents the heterogeneity of phase-activity patterns. Thus, these phase-related activity-based cell classes are bound to overcluster, suggesting that respiratory-related neurons form a continuum of patterns, rather than a set of discrete activity-based classes. Cell clustering applied to putative axonal rather than somatic units also indicates a continuum of activity-based cell classes (Supplementary Fig. 4).

## Neural trajectories target the offset of inspiration

Recording of simultaneous neural activity allows for the quantification of low-dimensional, coordinated activity patterns across the population of recorded neurons. The low-dimensional subspace that contains the majority of the neural variance is referred to as a neural manifold^3,47^, while the evolution of the low-dimensional activity through that subspace is referred to as its latent trajectory.

**Fig. 3:**
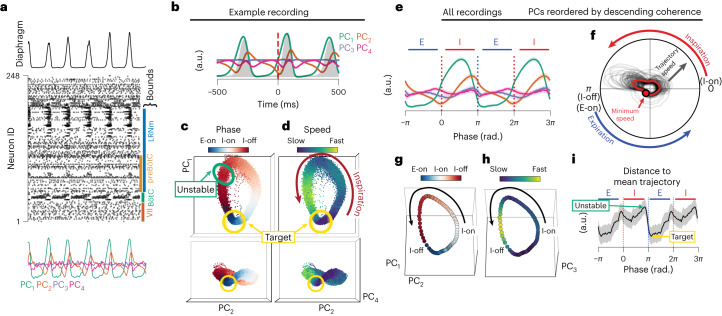
Low-dimensional manifold underlies respiratory neural activity. **a**, Example integrated diaphragm (top), raster (middle) for 248 simultaneously recorded units, with rostral–caudal boundaries of landmark medullary regions, and four (for illustration) leading PCs (bottom) that decompose the neural activity. **b**, Breath-aligned average PCs from **a**. **c**, Projection of the neural population into the space of the PC1, PC2 and PC4 for 10 min of recording time. PC3 is not shown as it is not coherent with the breathing cycle for this recording. Each dot is the three-dimensional position of these three PCs in a 5-ms time bin. Color represents the breathing phase. Inspiration onset occurs where dots are white; offset occurs where dots transition from red to blue. Unstable and target regions correspond to maximum and minimum variance in the trajectory as shown in **i**. **d**, As in **c**, but color represents the instantaneous temporal derivative of the trajectory through the PC space. **e**, PCs are reordered by respiratory coherence and average phase aligned values of the four (for illustration) most coherent PCs across all recordings are shown. Zero and 2*π* are diaphragm onset; *π* and 3*π* are diaphragm offset. PC color as in **a**. Shaded region is mean ± s.e.m. **f**, Angular plot of trajectory speed (radial axis) as a function of breathing phase (angular axis) for all recordings. Individual recordings in black, mean across recordings in red. Top half of circle is inspiration; bottom half is IBI. Minimum speed across all recordings is marked. **g**, Average PC trajectory in the coherence reordered space across all recordings colored by phase as in **c**. **h**, Same as **g** colored by trajectory speed. Each dot is a $\frac{\pi }{50}$ radian bin. **i**, For each recording, mean trajectory through the PC space was computed. Then, for each breath, the Euclidean distance to that mean trajectory was computed as a measure of breath-to-breath variability in latent trajectory. Distance to mean trajectory was then averaged across each breath and recording as a function of phase. Shaded region is mean ± s.e.m. Source data

**Extended Data Fig. 4:**
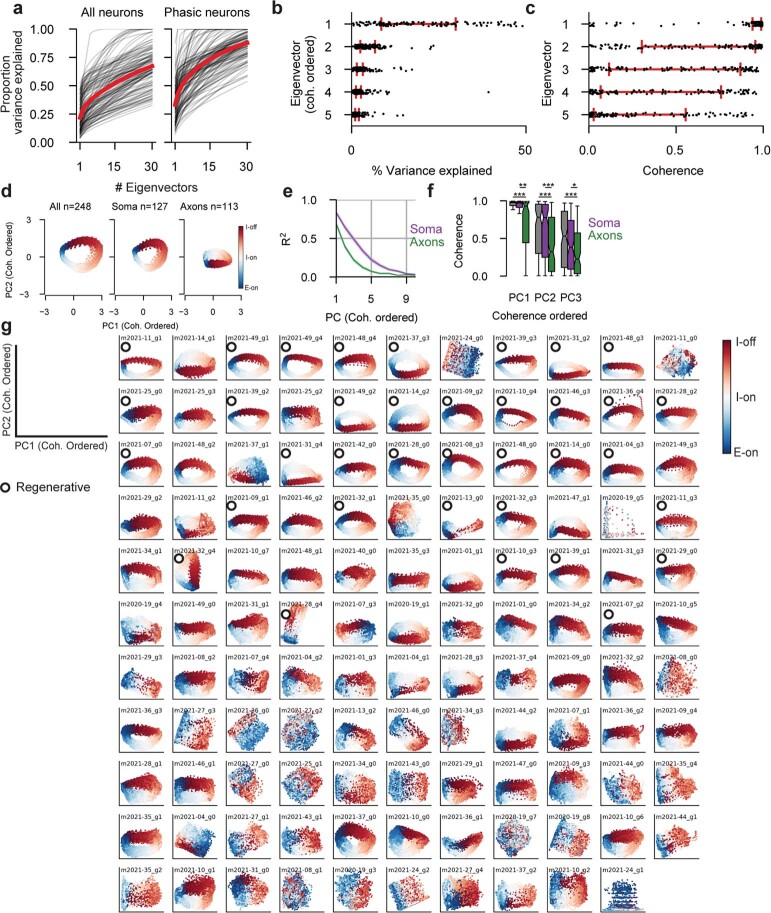
PCA decompositions of simultaneously recorded neural populations. (**a**) Cumulative explained variance as a function of number of PCs included. Black lines are individual recordings, red lines are mean across all recordings. Left is PCA decompositions which include all neurons, right is PCA decompositions that include only phasic neurons (*C*_*lb*_ > 0.1). (**b**) We reorder the PCs not by their eigenvalue but by the coherence of the PC with breathing in order to compare across recordings. The percent variance explained for eigenvectors in descending order of coherence is shown. After re-ordering the PCs, on average the eigenvectors decrease in their explained variance ratio monotonically, suggesting that the coherence re-ordered PCs still capture a large amount of the variance of the neural population in the leading PCs. Black dots are individual recordings, red error bars are median +/- IQR (**c**) Similar to (b) we show the coherence value for the leading five eigenvectors (now ordered by eigenvalue). On average, the leading eigenvectors are most coherent with breathing. (**d**) Example trajectories for a recording using all units (left), only somatic units (middle), or only axonal units (right). (**e**) *R*^2^ between somatic only (purple) or axonal only (green) PC projections with all recorded units. Shaded region is mean +/- S.E.M. (**f**) Coherence of the first three PCs with breathing for all recorded units (grey), somatic only units (purple), or axonal only units (green) Soma only PCs are not less coherent than full PCs, but axonal PCs are (One-way RM ANOVA n = 114 recordings. PC1: F_(1.503,169.8)_=11.1, p = 0.002; PC2:F_(1.809,204.4)_=28.07, p < 0.0001; PC3: F_(1.652,186.7)_=11.55, p < 0.0001. Tukey’s multiple comparisons post-hoc test *p < 0.05,**p < 0.01 ***p < 0.001). Boxes are mean +/- 1 quartile, whiskers are +/- 1.5 IQR. (**g**) Projections of population activity onto the two most coherent PCs for all recordings. Many recordings exhibit similar rotational structure that is highly consistent. Further, most recordings show a strong mapping between position on the manifold and respiratory phase (red->blue). Circles indicate recordings that were also regenerative in the rSLDS models.

We first use principal components analysis (PCA) to decompose the high-dimensional activity of simultaneously recorded units (Fig. 3a–d and Extended Data Fig. 4). All units recorded simultaneously for a given recording (including somatic and axonal units from all locations on the probe) are included in a given PCA, but inclusion only of somatic units generally recapitulates results seen with the full dataset (Extended Data Fig. 4). Low-dimensional neural activity evolves through a consistent rotational trajectory in the principal component (PC) space, and the location along that trajectory is highly correlated with the respiratory phase (Fig. 3b,c,e,g and Supplementary Videos 3 and 4). These trajectories show conserved temporal evolution such that the trajectory is fastest (that is, the population activity changes most quickly) during the inspiration-off/expiration-on transition (Fig. 3d,h). To compare PCs across recordings, we reorder the PCs by coherence such that the leading PC is the most coherent with respiration, regardless of the variance accounted for (Extended Data Fig. 4). We compute the average low-dimensional trajectory across all recordings (Fig. 3e–i) by first reordering the PCs by their respiratory coherence, and then normalizing the time domain to phase. We visualize the first four PCs for illustration, but some respiratory coherence is observed even in higher-order PCs (Extended Data Fig. 4). Ordered PC_1_ exhibits pre-inspiratory and inspiratory activity, PC_2_ augments during inspiration and peaks at the offset of inspiration, and PC_3,4_ exhibit more complex, but consistent, relationships with phase. Both trajectory speed (which quantifies rate of change of neural state) and distance to mean trajectory (which quantifies the breath-to-breath variability of neural state) are maximal before offset of inspiration, and minimal just after offset of inspiration (Fig. 3f,i). This suggests that the peak of inspiration represents an unstable region of the latent space, and the offset of inspiration resembles an attractive ‘target’. Such a target represents that the neural configuration of inspiration-off is most consistent across breaths. This low-dimensional landscape is conserved across recordings and animals (Extended Data Fig. 4).

## Dynamical systems modeling of VRC population activity

The above PCA inherently treats all points in time as independent and does not incorporate temporal dynamics of the neural activity. Therefore, we estimate latent linear dynamics from the high-dimensional observations (that is, the activity of the recorded neural population) using a recently developed recurrent switching linear dynamical system (rSLDS)^48^. This model allows for multiple sets of linear dynamical systems to govern the latent evolution (‘switching’), and the dynamics to be employed at any one point in time are determined by the current latent state (‘recurrent’). Thus, we approximate the underlying nonlinear dynamical system with discrete, intersecting linear systems.

**Fig. 4:**
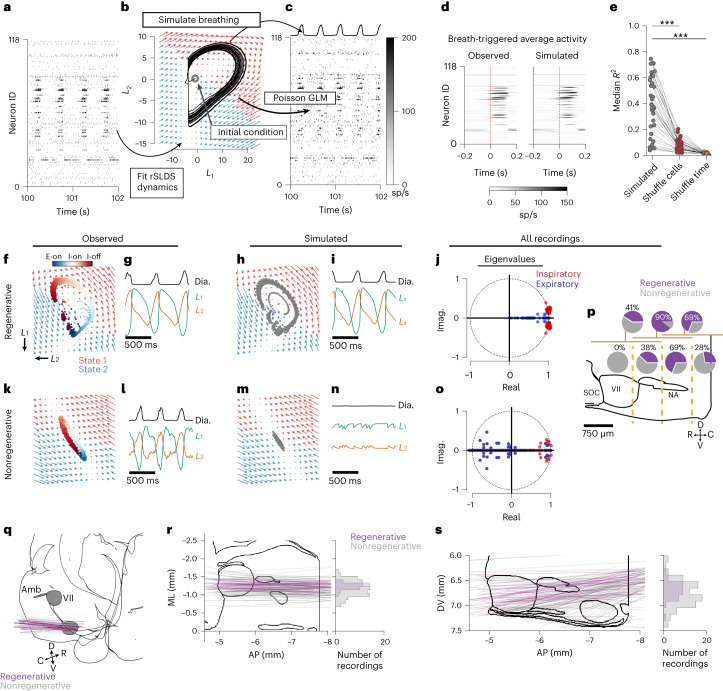
Inspiration and expiration follow alternating linear dynamical systems. **a**, Two seconds of recorded neural activity across 118 VRC units. Color scale is shown in **c**. **b**, Flow fields and simulated trajectories as determined by the rSLDS model. Arrow color indicates underlying dynamics state. **c**, Poisson GLM simulated spike rate of the neural population. Top: simulated diaphragm activity. **d**, Breath onset-aligned spike rate averages for all 118 recorded (left) and simulated (right) populations. **e**, Median *R*^2^ compared to observed breath triggered spike rates between regenerative recordings. Each point is median *R*^2^ of all units in a given recording. Simulated *R*^2^ are greater than controls in which neuron identity (shuffle cells) or time bins (shuffle time) are shuffled. (***P < 0.001 two-sided paired *t*-test; simulated versus shuffle cells: *t* = 9.37, *P* = 3.4 × 10^−11^, simulated versus shuffle time: *t* = 9.67, *P* = 1.5 × 10^−11^, *n* = 37). **f**, The observed trajectory through the 2D latent space is shown as dots (10-ms time bins). Plot has been rotated for visualization. **g**, Observed diaphragm activity (black) and the two latent variables (green and orange) over ~1 s. **h**, The inferred dynamics (flow fields identical to **f**) simulate evolution through the latent space. Gray dots indicate simulated latent. **i**, As in **g** for simulated latent evolution. **j**, Inferred latent dynamics were active during inspiration or expiration. The real and imaginary components of the eigenvalues are shown for the dynamics matrices that govern either inspiration (red) or expiration (blue). Dashed line is the unit circle. **k**–**o**, As **f**–**j** for recordings that did not simulate rhythmic diaphragm activity. **p**, regenerative rSLDS models were given access only to rostrocaudally restricted subsets of recorded units. Vertical yellow dashed lines indicate borders of the four anatomical subsets imposed. Pie charts indicate percentage of recordings that maintain regenerative capability (purple) with indicated subsets. Bottom row includes only one subset; top row includes units of two neighboring subsets indicated by dark yellow horizontal lines. **q**–**s**, Three-dimensional (**q**) and two-dimensional (**r** and **s**) projections of the probe tracks that resulted in regenerative (purple) or nonregenerative (gray) dynamics. Marginal histograms show the mediolateral (**r**, right) and dorsoventral (**s**, right) location of the probe midpoint.VII, facial motor nucleus; Amb, nucleus ambiguus. Source data

**Extended Data Fig. 5:**
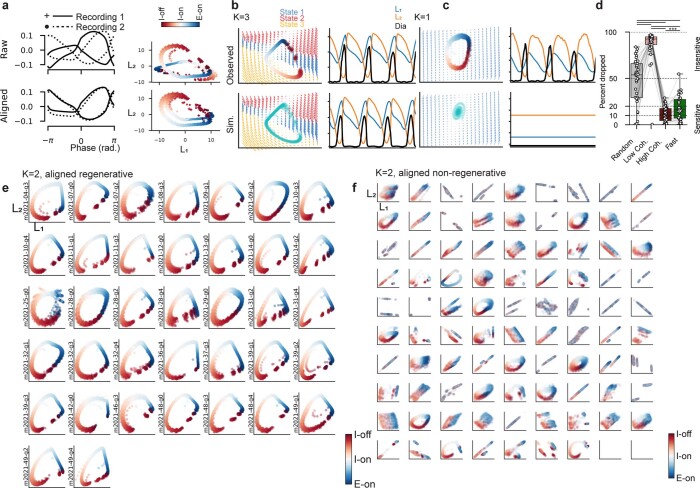
Recurrent linear dynamical systems uncover conserved latent dynamics that switch between two states if sufficient populations are recorded to recreate regenerative dynamics. (**a**) The latent structure of 2 separate recordings may have similar qualitative characteristics but differ in the permutation and scaling of the latent axes (top). We linearly re-align the two recordings (see Methods) such that the trajectories overlap one another (bottom). Left shows the phase aligned average of the two latent components for two recordings before (top) and after (bottom) linear alignment. The phase-colored trajectories through the unaligned (top) and aligned (bottom) latent spaces. Dots are 10 ms time bins. (**b**) Increasing the number of states (that is, partitions of the latent space with different dynamics) for the rSLDS does not change the qualitative structure of the latent trajectories and does not increase the number of recordings that are regenerative (37 for *K* = 2, and for *K* = 3). (**c**) Allowing no partitions (*K* = 1) of the latent space prevents any recording from being regenerative (example shown top), despite oscillatory structure in the observed data. Rather, single state LDSs form decaying spirals. Panel organized as in Fig. 4f-i. (**d**) Percent of units that can be removed while maintaining regenerative simulated diaphragm activity. Sequential removal of units in order of respiratory coherence or firing rate: random, lowest coherence first, highest coherence first, highest firing rate first (One-way RM ANOVA F_(2.337,84.13)_=318, p < 0.0001; Tukey’s multiple comparisons post-hoc test lines are p < 0.0001, ***p < 0.001; n = 37 recordings). Box is median +/- one quartile, whiskers are 1.5 * IQR. **e**) The trajectories of all regenerative recordings, and (**f**) non-regenerative recordings, aligned to a reference recording. Markers are colored by phase as in (a).

We fit two-state, two-dimensional rSLDS (Methods) to compute the latent dynamics of the neural population (Fig. 4a,b and Extended Data Fig. 5). As with the PCA, all simultaneously recorded units (including putatively somatic and axonal units from all anatomical locations on the probe) are included to fit the rSLDS. We then simulated the latent dynamics (Fig. 4b) and resultant neural activity (Fig. 4c) by supplying the dynamics matrices as inferred from the experimental data and visualized by the flow fields in Fig. 4b. Simulated firing rates were estimated using a Poisson generalized linear model (GLM) with the latent state as inputs. Lastly, we use support vector regression to predict the diaphragm activity from the two-dimensional latent state (Fig. 4c). This allows us to simulate the entire recorded population and diaphragm activity from only the two-dimensional latent state. By aligning the simulated firing rates to the simulated diaphragm onsets, we can well recreate the breath-averaged activity of the recorded VRC population (Fig. 4d,e). We found that the dynamics fit from a recording can either be ‘regenerative’ (Fig. 4f–j), in that the dynamics matrices (that is, flow fields) alone are sufficient to recreate oscillatory latent state evolution and subsequent rhythmic simulated diaphragm activity (Fig. 4h,i), or nonregenerative (Fig. 4k–n). In the nonregenerative case, simulations could not produce oscillations or rhythmic simulated diaphragm activity, despite oscillations in the observed latent dynamics (Fig. 4k,l). Dropout analysis of the regenerative recordings determined that regeneration relied on inclusion of sufficient populations of highly coherent units (Extended Data Fig. 5d). Thus, if a model was nonregenerative, it means that the neural population sampled did not observe enough coherent units to generate oscillatory dynamics. Units with high firing rates are also important for regenerative dynamics, but these fast units are more likely to be highly coherent (Spearman correlation coherence versus average firing rate; *R* = 0.43, *P* = 0, *n* = 15,299). Models with only 1 state (*K* = 1, that is, not switching^48^) could never be regenerative (Extended Data Fig. 5), but rather formed decaying spirals. Models with three states were no more likely to be regenerative than models with two states (*n* = 37/116 regenerative for *K* = 2, 37/116 regenerative for *K* = 3; Extended Data Fig. 5).

We then investigate the properties of the dynamics matrices as inferred by the rSLDS models. The two inferred states of regenerative recordings are clearly restricted to either the inspiratory or expiratory phase of the breath. That is, the neural population follows one set of dynamics during inspiration, and another during expiration. For the regenerative recordings, the dynamics were strikingly similar across recordings (Extended Data Fig. 5e). By examining the eigenvalues of the *A* matrix for each state (Fig. 4j), we find that all inspiratory dynamics were governed by spirals (37/37 recordings), and these spirals were mostly unstable (28/37 recordings). Expiratory dynamics rarely exhibited rotations (3/37 recordings) and were governed by stable nodes (32/37 recordings) or saddles (5/37 recordings). These dynamical regimes intersected to generate alternation between these two states, which approximates an underlying nonlinear system.

To test whether rostrocaudal subregions of the VRC were sufficient to recapitulate oscillatory dynamics, we refit and resimulated all the regenerative recordings but systematically removed subsets of the population along the rostrocaudal extent of the recording. Models with access to the caudal or rostral extremes of the VRC were less likely to be regenerative than those with access to the middle sections that contain the preBötC and BötC (Fig. 4p).

In contrast, nonregenerative recordings showed no clear patterns in their dynamics across recordings and states (Fig. 4o and Extended Data Fig. 5e). We found that whether the recording was regenerative or not depended primarily on the mediolateral anatomical location of the recording. If the recording was restricted to the center of the VRC, where many respiratory tuned neurons are located, it was more likely to be regenerative than at the borders of the VRC (Fig. 4q–s).

## Opioids alter timing, not rotational dynamics, of population activity

**Fig. 5:**
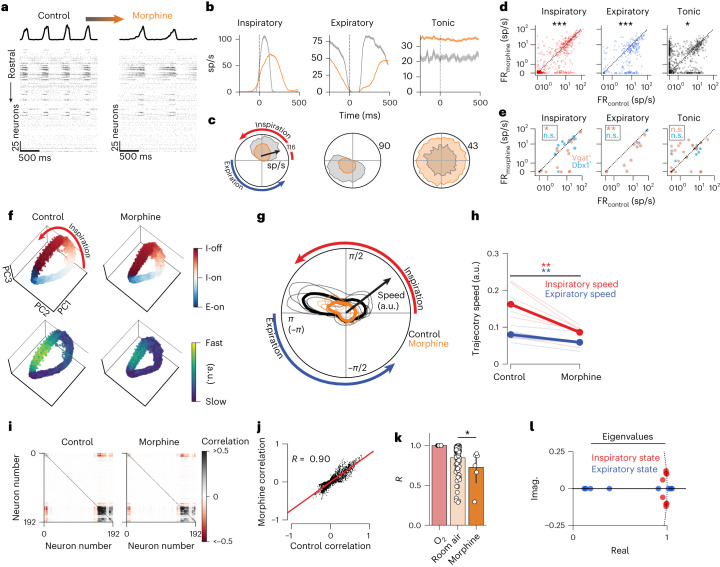
Opioids cause temporal restructuring of respiratory neural activity, but do not alter rotational dynamics. For all panels, gray is control and orange is morphine. **a**, Example rasters and diaphragm activity before (left) and after (right) intraperitoneal administration of 150 mg kg^−1^ morphine. **b**,**c**, Breath onset- (**b**) and phase- (**c**) aligned average activity of three example units in control and after morphine administration. Top half of circle is inspiration; bottom half is expiration. Mean firing rate ± s.e.m. is shown. sp/s, spikes/second. **d**, Firing rates of inspiratory (red, *n* = 594), expiratory (blue, *n* = 245) and tonic (black, *n* = 771) units before (*x* axis) and after (*y* axis) morphine administration. Two-sided Wilcoxon rank sum test: *P* = 7.0 × 10^−5^, 4.97 × 10^−15^ and 0.020, respectively. **e**, Firing rates of optogenetically tagged units (blue: Dbx1^+^ *n* = 23, orange: Vgat^+^ *n* = 57) separated by respiratory firing pattern (two-sided Wilcoxon rank-sum test; Vgat_tonic_ *P* = 0.99, Vgat_insp_ *P = 0.01, Vgat_exp_ **P = 0.002, Dbx1_tonic_ *P* = 0.30, Dbx1_insp_ *P* = 0.57, Dbx1_exp_ *P* = 0.50). **f**, Low-dimensional population projections onto the leading three PCs. Each dot is a 5-ms bin. Top row color indicates phase; bottom row color indicates trajectory speed. **g**, Angular plot of trajectory speed (radial axis) as a function of breathing phase (angular axis) for all recordings. Individual recordings are thin traces; means across all recordings are thick traces. **h**, Average speed during inspiratory phase (red) or expiratory phase (blue) in control versus morphine (two-sided paired *t*-test expiratory *P* = 0.007, inspiratory *P* = 0.002, *n* = 6). Individual recordings are thin traces; means are thick traces. Trajectory speed units are arbitrary. **i**, Correlation of each pair of recorded units in control (left) and morphine (right), ordered by ward clustering. **j**, Correlation value of each pair of units before (*x* axis) and after (*y* axis) morphine administration. Each dot is a pair of units. **k**, Linear fit (*R* value) of the pairwise correlation values in O_2_ compared to room air and during morphine administration. Each dot is a recording; error bars are 95% confidence interval. We consider room air as a control change in correlation structure as compared to O_2_. Changes in population correlation structure after morphine administration are modestly different than those observed in room air (two-sided Mann–Whitney *U* test *P* = 0.026, *n*_roomair_ = 116, *n*_morphine_ = 6). **l**, Real and imaginary components of the eigenvalues for the dynamics matrices that govern the inspiratory and expiratory states in the rSLDS models fit during OIRD. Only recordings in which the dynamics were regenerative during control eupnea (*n* = 4) are shown. Dashed line is the unit circle. Source data

**Extended Data Fig. 6:**
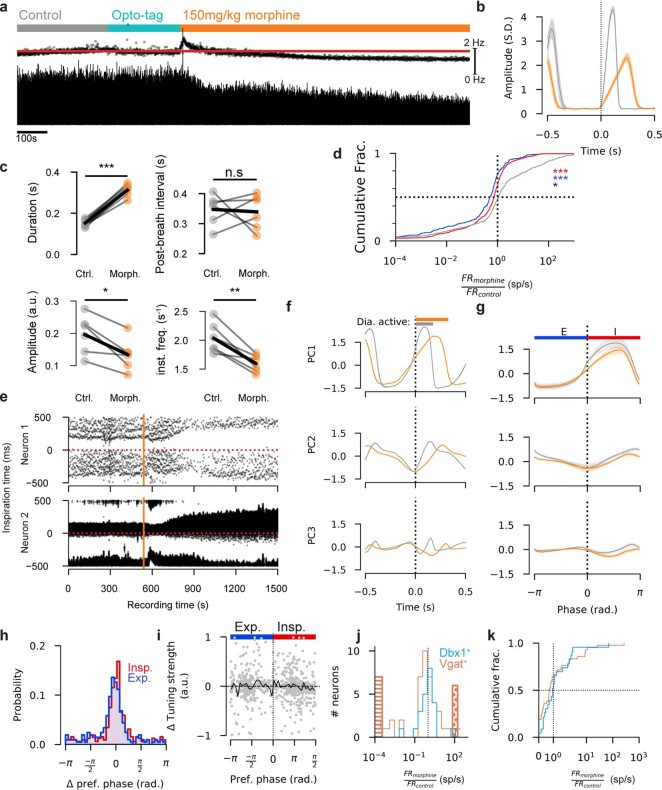
Neural population responses to morphine administration. (**a**) Example integrated diaphragm trace before and after morphine administration. Top scatter is instantaneous respiratory rate. Red line is average respiratory rate in control period. Scale bar for respiratory rate is right (0–2Hz). (**b**) Average breath onset aligned diaphragm activity in control and morphine. Shaded regions are mean ± S.E.M. (**c**) Changes in diaphragm amplitude, burst duration, respiratory frequency, and post-breath interval as a result of morphine administration for all recordings (n = 6) (two-sided paired t-test; duration ***p = 1.8e-5; post-burst interval p = 0.82; *amplitude p = 0.01; **instantaneous frequency p = 0.005) (**d**) Cumulative probability distribution of the ratio of firing rates in morphine to firing rates in control, for all recorded neurons. On average, inspiratory (n = 594) and expiratory (n = 245) units have reduced firing rates, while tonic units (n = 771) have higher firing rates (two-sided Wilcoxon rank-sum test p = 7.01e-5, p = 4.97e-15, p = 0.020 respectively) (**e**) Breath onset aligned rasters for example expiratory (top) and inspiratory (bottom) units over the course of morphine administration. X-axis is time in the recording of a given breath, y-axis is breath onset aligned spike time. Red dashed horizontal line is breath onset time. Each column is a breath, and each dot is a spike. Morphine is administered at the vertical orange line. (**f**) Breath onset aligned average of the first 3 PCs of neural population in control and morphine for an example recording. Morphine dramatically slows the evolution of the population activity. (**g**) Phase-aligned average PCs of neural population across all recordings. Shaded region is mean ± S.E.M. Phasic structure of the PCs is preserved in morphine, with a slight reduction in amplitude of PC1. (**h**) Histogram of change in preferred phase of all recorded neurons. (**i**) Change in respiratory tuning strength as a function of preferred phase. Each dot is a neuron, solid black line is the mean change in tuning strength. Units with fewer than 1 spike per breath are excluded in h and i. (**j**) Histogram of firing rate ratio for optogenetically tagged units. Units that are completely silenced in morphine are shown in horizontal hatching; units that are silent in control and not in morphine are shown in dot hatching. (**k**) Cumulative distribution of FR_ratio_ for optogenetically identified neurons.

We next sought to quantify network-wide activity changes in response to systemic opioid administration. To investigate network-wide neural reconfiguration during OIRD, we administered a single dose of morphine (150 mg kg^−1^, 4 Vgat^Cre^;Ai32 mice, 2 Dbx1^CreERT2^;Ai32 mice) during recordings from the VRC. Respiratory rate decreased by an average of 21% (± 9% standard deviation (s.d.)), and inspiratory duration increased by an average of 105% (± 22% s.d.) (Extended Data Fig. 6). An example raster of 262 simultaneously recorded neurons and associated diaphragm activity are shown before and after morphine administration (Fig. 5a). Gas supplied was 100% O_2_, and we include both somatic and axonal units in these analyses.

We first determined the extent to which morphine altered the activity of single neurons of the VRC. We found that morphine does not unilaterally reduce the firing rate of neurons, but instead induced coordinated changes across the entire population of VRC neurons (Fig. 5b–e). The duration of spiking in individual neurons increased and spike rates changed for many neurons, but the phasic patterns were preserved (Fig. 5b,c and Extended Data Fig. 6). On average, inspiratory and expiratory units show reduced firing during morphine administration, while tonic units are activated. Of the 1,610 units recorded after morphine administration, 57 were identified as Vgat^+^ (inhibitory) and 23 as Dbx1^+^ (excitatory). On average no change was observed in Dbx1^+^ units. Inspiratory and expiratory inhibitory neurons showed reduced firing, while presumably distinct populations of tonic inhibitory neurons were either recruited or silenced (Extended Data Fig. 6j,k).

**Extended Data Fig. 7:**
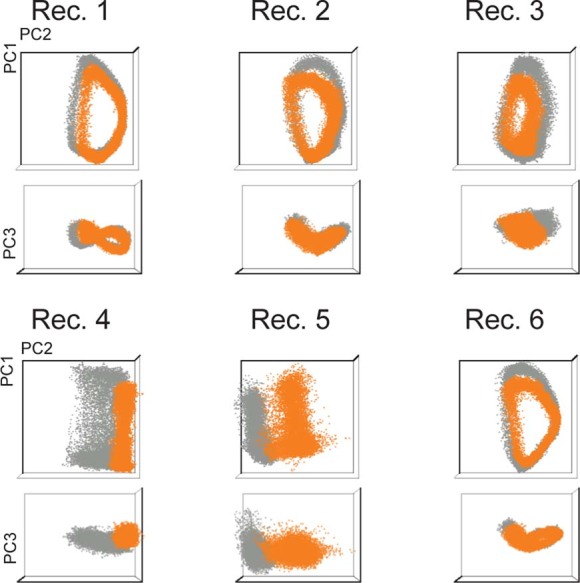
Changes to low-dimensional structure after opioid administration. Projections of the population activity onto the leading three PCs for all recordings in which morphine was administered. Grey dots show control period, orange dots show after morphine administration. Recordings which show rotational activity are largely preserved in morphine, while those with little rotational activity show larger changes suggesting that morphine acts not to disrupt the oscillatory behavior of the population, but rather affects the non-oscillatory modes.

To determine the extent to which coordinated population activity changes during OIRD, we examined the neural manifolds before and after morphine administration. We found that rotational PC trajectories of the neural population were qualitatively similar across conditions (Fig. 5f and Extended Data Figs. 6g and 7). The relationship between location in the PC space and breathing phase was conserved (Fig. 5f), indicating that the underlying latent structure of the population remained largely unchanged. The inspiration-off target was preserved during opioid administration (Fig. 5f). Surprisingly, morphine slowed the evolution of the trajectory through PC space (Fig. 5g and Supplementary Video 5). This slowing occurred at most phases of the respiratory cycle, but most dramatically during the second half of inspiration (Fig. 5g,h). By examining the breath aligned averages of the PCs we saw that morphine slowed the latent temporal evolution of the PCs, but the phasic relationship between these PCs was preserved.

**Extended Data Fig. 8:**
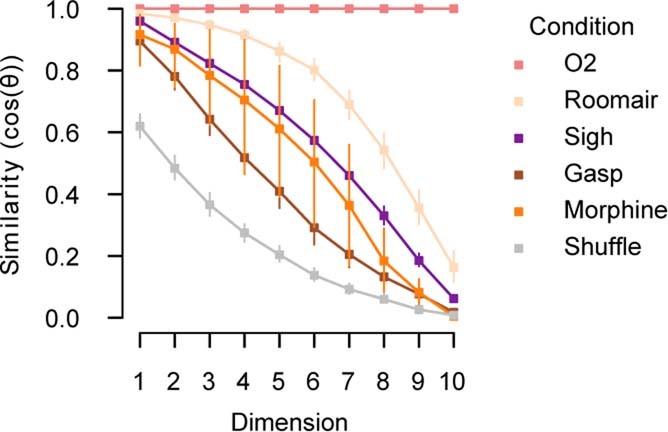
Low-dimensional structure is disrupted during gasping. We fit PCA separately during different conditions and compute the principal angles between the subspaces spanned by the leading eigenvectors for each condition. Similarity (defined as the cosine of the principal angles) between the leading dimensions of the PCA decomposition for each condition as compared to control (100% O_2_). Unsurprisingly, the similarity is highest for the room air condition. Sighs and OIRD (morphine) are next similar, and gasps are least similar. To compute the lower bound on similarity, we shuffle the entries of the eigenvectors for O_2_ and compute the principal angles between the shuffled space and the original space (gray). Markers are median and lines are +/- IQR (n_O2_ = 125, n_sigh_ = 121, n_gasp_=38, n_morphine_=6, n_roomair_ = 116, n_shuffle_ = 125).

We next computed the pairwise zero-lag correlations of spike rates for all neurons in baseline (100% O_2_) and after morphine administration. We found that the change in correlation structure during OIRD was more pronounced than the change observed when exposed to room air (Fig. 5i–k), primarily in recordings that did not exhibit rotations during baseline. We further quantified the changes in low-dimensional structure by separately computing the PCA decompositions in control and in morphine, and then comparing the subspaces spanned by the leading eigenvectors of those decompositions^49^. We quantified the similarity between these subspaces using principal angles (Methods) and found that the subspaces largely overlapped, indicating overall conservation of the low-dimensional population structure in OIRD (Extended Data Fig. 8). Lastly, to confirm that the rotational dynamical structure was not disrupted during OIRD, we refit the rSLDS model only on data recorded after morphine administration, for the recordings that were regenerative during normal breathing (*n* = 4). We found that spiral sources and stable/saddle nodes continued to govern inspiration and expiration, respectively. (Fig. 5l and Supplementary Fig. 5).

## Sighs are inspiratory excursions along neural manifold

Sighs are intermittent, large amplitude breaths that occur at regular intervals^50^. Sighs are critical for maintaining blood gas homeostasis by preventing atelectasis; loss of this behavior is fatal^51^. As sighs are known to cause reconfiguration of VRC activity, we sought to determine how VRC population activity was modulated during sighing.

**Fig. 6:**
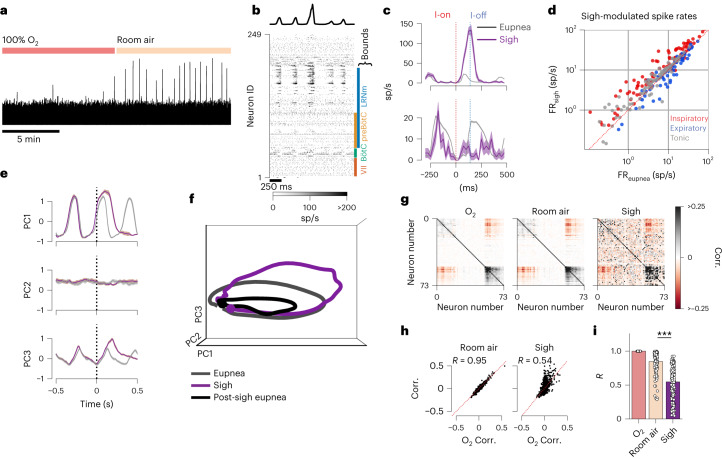
Sighs are inspiratory excursions that disrupt subsequent breaths. **a**, Integrated diaphragm trace over 20 min shows increased frequency of sighs during room air presentation. **b**, Example spike raster and diaphragm activity before and after a sigh. Approximate rostral–caudal boundaries of some landmark medullary regions are shown. **c**, Breath onset-aligned average activity of two example units (mean firing rate (FR) ± s.e.m.) for eupnea (gray) and sighs (purple). Firing rate of top unit increases and bottom unit decreases during the sigh. sp/s, spikes/second. **d**, Firing rates of all units recorded in **b** for eupnea (*x* axis) and sighs (*y* axis). Each dot is a recorded unit. Color indicates preferred phase of firing during eupnea: inspiratory (red), expiratory (blue) or tonic (gray). **e**, Breath onset aligned average of the leading three PCs in eupnea (gray) and sighs (purple) for an example recording. Shaded region is mean ± s.e.m. **f**, Average population trajectories through PC space for the recording in **e** for eupnea (gray), sighs (purple) and the breath after a sigh (black). **g**, Correlation values for all pairs of units during eupnea in O_2_, room air, and during sighs. Red is strong negative correlations; black is strong positive correlations. **h**, Correlation (Corr.) value of each pair of units during O_2_ presentation (*x* axis) against those during room air presentation (*y* axis, left) and during sighs (*y* axis, right). **i**, Linear fit (*R* value) of the pairwise correlation values in O_2_ compared to room air and sighs. Each dot is a recording; error bars are 95% confidence interval. We consider room air as a control change in correlation structure as compared to O_2_. Changes in population correlation structure during sigh are different than those observed in room air (two-sided Mann–Whitney *U* test ***P = 1 × 10^−22^, *n*_recs___roomair_ = 116, *n*_recs_sigh_ = 121). Source data

**Extended Data Fig. 9:**
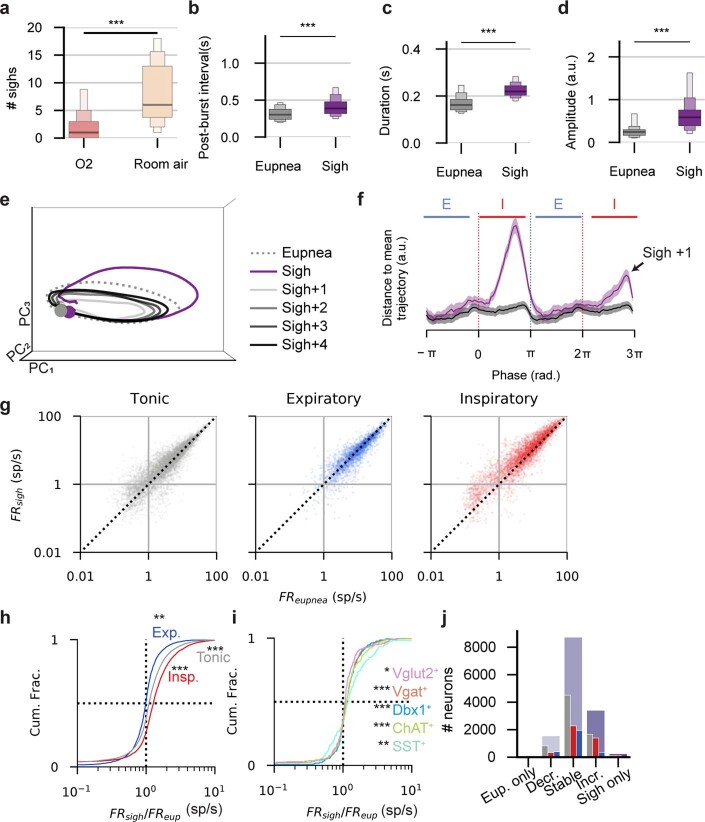
Neural population changes during sighing. (**a**) Average number of sighs observed in 100% O_2_ and in room air across all recordings with both room air and O2 presentations (two-sided Wilcoxon rank-sum test ***p = 1.3e-14, n =116 recordings). (**b**) Average post-burst interval (time from breath cessation to onset of next breath), (**c**) breath duration, and **(d**) breath amplitude are increased during sighs (two-sided paired t-test ***p < 0.001, n = 126 recordings; (b) p = 2.0e-22, (c) p = 3.6e-22, (d) p = 3.5e-21). (**e**) Mean neural PC trajectories as in Fig. 6f, but including the first through fourth eupnea after the sigh. Recovery to a normal eupneic trajectory takes several breaths after a sigh. (**f**) Distance to mean eupnea trajectory as in Fig. 3i, averaged across all recordings and breaths. Shaded region is mean ± S.E.M. Distance from sigh to eupnea shown in purple. (**g**) Firing rates for all units across all recordings during eupnea (x-axis) and sighs (y- axis). Each dot is a unit, units are separated by their preferred phase in eupnea. Somatic and axonal units are shown (**h**) Cumulative probability distribution of the ratio of firing rates in sigh to firing rates in eupnea (FR_ratio_), for all recorded units. All units are right shifted (that is, recruited) during sighs (two-sided, 1-sample t-test on log firing rate ratio; expiratory: n = 2,905 units, p = 0.002; inspiratory n = 4,453 units, p = 2.26e-233; tonic n = 7,716 units, p = 7.3e-90). (**i**) As in (h) for neurons of optogenetically identified as indicated by color. All subgroups units are on average recruited during a sigh (two-sided 1 sample t-test on log firing rate ratio; Vglut2^+^ n = 192, *p = .034; ChAT^+^ n = 156,***p = 6.6e-6; Vgat^+^ n = 334, ***p = 2.33e-6; Dbx1^+^ n = 346, ***p = 6.04e-12; SST^+^ n = 106, **p = 0.003). (**j**) Neurons are categorized by the FR_ratio_ as being eupnea only (FR_ratio_<0.2), sigh decreasing (0.2 ≤ FR_ratio_ < 0.7), stable (0.7 *≤* FR_ratio_ < 1.5), sigh increasing (1.5 ≤ FR_ratio_ < 5), or sigh only (5 ≥ FR_ratio_). The total number of neurons in each category are shown in purple, and are subdivided by inspiratory, expiratory, and tonic. Boxen plots are median, boxes are median ± first, second, and third quantile respectively.

A sigh is exemplified by increased diaphragmatic activation and a prolonged period of apnea after the sigh (Extended Data Fig. 9). In this experimental preparation we could evoke sighs by presenting room air (21% O_2_) instead of 100% O_2_ (Fig. 6a). Sighs show pronounced increase in firing rates of inspiratory neurons throughout the VRC (Fig. 6b–d and Extended Data Fig. 9).

Examination of the low-dimensional trajectories of eupnea and sighs shows that sigh trajectories largely overlap with eupnea during the expiratory and pre-inspiratory phase, exhibit an augmented excursion during the inspiratory phase and target the same post-inspiratory region that was observed during eupnea (Fig. 6e,f and Supplementary Video 3). This altered trajectory is consistent across animals and recordings (Extended Data Fig. 9f). The trajectories of the subsequent breath after the sigh trace out the same shape as a eupnea in the PC space, but with smaller radii, indicating the neural population is incompletely recruited in the breaths following a sigh (Fig. 6f). The recovery to normal eupnea after a sigh takes several breaths (Extended Data Fig. 9e).

We show that correlated activity across the VRC population is altered during sighs by comparing the pairwise correlations between eupnea in 100% O_2_ with eupnea in room air and with sighs (Fig. 6g–i). The pairwise correlations are altered during sighs in comparison to eupnea in room air, but the principal angles between the eupneic and sigh low-dimensional projections show that the subspaces are largely overlapping (Extended Data Fig. 8), indicating a minor and temporary disruption of VRC manifolds.

## Gasps collapse rotational dynamics into ballistic trajectories

**Fig. 7:**
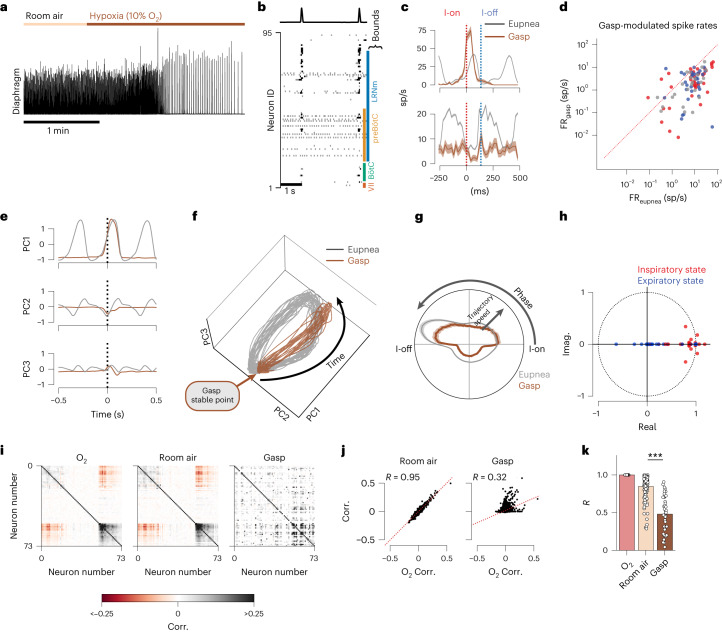
Gasps collapse latent orbits into ballistic, all-or-none efforts. **a**, Integrated diaphragm activity illustrating the transition to gasping during hypoxia presentation. **b**, Example spike raster and diaphragm activity during a period of gasping. Approximate rostral–caudal boundaries of some landmark medullary regions are shown. **c**, Breath onset-aligned average firing rates of two example units. Maximum firing rate (FR) of top unit increases and bottom unit decreases during gasping. Shaded region is mean FR ± s.e.m. sp/s, spikes/second. **d**, Firing rates of all units recorded in **b** for eupnea (*x* axis) and gasps (*y* axis). Each dot is a recorded unit. Color indicates preferred phase of firing during eupnea: inspiratory (red), expiratory (blue) or tonic (gray). **e**, Breath onset-aligned average of the leading three PCs in eupnea (gray) and gasps (brown) for the recording in **b**. **f**, Population trajectories through PC space for the recording in **b** for eupnea (gray) and gasps (brown). **g**, Trajectory speed (radial axis, a.u.) as a function of breathing phase (angular axis) for eupnea (gray) and gasps (brown). Top of circle is inspiratory effort; bottom of circle is IBI. Shaded region is mean ± s.e.m. across all recordings. **h**, Real and imaginary (Imag.) components of the eigenvalues of the dynamics matrices that govern the inspiratory and expiratory states in the rSLDS models fit during gasping. Only recordings in which the dynamics were regenerative during control eupnea (*n* = 11) are shown. Dashed line is the unit circle. **i**, Correlation (Corr.) values for all pairs of units during eupnea in O_2_, room air, and during gasps. Red is negative correlations; black is positive correlations. **j**, Correlation value of each pair of units during O_2_ presentation (*x* axis) against those during room air presentation (*y* axis, left) and during gasps (*y* axis, right). **k**, Linear fit (*R* value) of the pairwise correlation values in O_2_ compared to room air and gasps. Each dot is a recording; error bars are 95% confidence interval. We consider room air as a control change in correlation structure as compared to O_2_. Changes in population correlation structure during sigh are different than those observed in room air (two-sided Mann–Whitney *U* test ***P = 7.3 × 10^−14^, *n*_recs___roomair_ = 116, *n*_recs_gasp_ = 38). Source data

**Extended Data Fig. 10:**
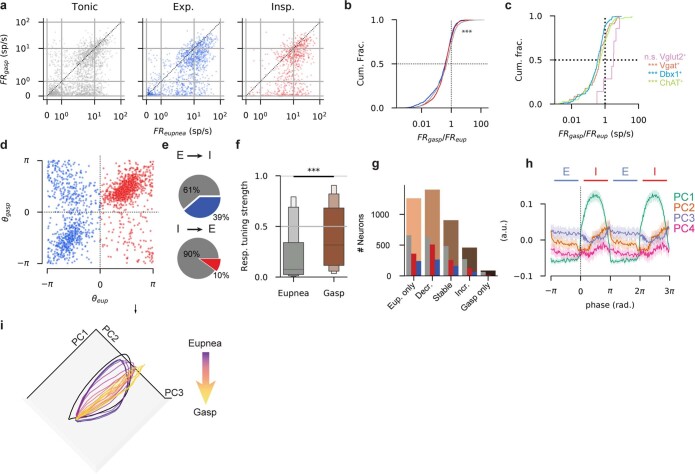
Neural population changes during gasping. (**a**) Firing rates for all units across all recordings during eupnea (x-axis) and gasps (y- axis). Each dot is a unit, units are separated by their preferred phase in eupnea. Somatic and axonal units are shown (**b**) Cumulative probability distribution of the ratio of firing rates in sigh to firing rates in eupnea (FR_ratio_), for all recorded neurons. All populations are left-shifted (that is, reduced firing rates) during gasping. (Two-sided 1-sample t-test on log firing rate ratio; tonic: n = 2,108 units, p = 2.12e-147; inspiratory n = 1,271 units p = 2.77e-124; expiratory n = 733 units p = 7.39e-79). (**c**) As in (b) for neurons of optogenetically identified genotype as indicated by color. (Two-sided 1 sample t-test on log firing rate ratio; Vglut2^+^ n = 7 units, p = 0.10; Dbx1^+^ n = 201 units, p = 7.12e-28; ChAT^+^ n = 104 units, p = 2.55e-7; Vgat^+^ n = 56 units p = 1.86e-5) (**d**) The direction selectivity *(L*) of each neuron (*θ*) is plotted as computed during eupnea (x-axis) and gasping (y-axis). Neurons along the diagonal show stable phase preference, while neurons off the diagonal show switching of direction selectivity. (**e**) 39% of expiratory units became inspiratory during the gasp, while 10% of inspiratory units became expiratory. (**f**) Distribution of respiratory tuning strength of all units during eupnea and gasping. On average units become more tuned to respiration during gasping. Boxes are median ± first, second and third quantile, respectively. (p = 0 Two-sided Wilcoxon rank-sum test. n = 4,112 units) (**g**) Neurons are categorized by the FR_ratio_ as being eupnea only (FR_ratio_<0.2), gasp decreasing (0.2 ≤ FR_ratio_ < 0.7), stable (0.7 ≤ FR_ratio_ < 1.5), gasp increasing (1.5 ≤ FR_ratio_ < 5), or gasp only (5 ≥ FR_ratio_). The total number of units in each category are shown in brown, and are subdivided by inspiratory, expiratory and tonic. In general, neurons are de-recruited during the gasp. (**h**) Breath-aligned average first 3 principal components for an example recording during eupnea and gasping. Pre- and post- inspiratory population activity is largely lost during gasping. (**i**) The collapse from rotational to ballistic dynamics is gradual. Each line is the average trajectory in sequential 10s periods during the transition from eupnea (purple) to gasping (yellow). The black trajectory is the average eupneic trajectory prior to hypoxia exposure.

Under severely hypoxic conditions, the respiratory network generates auto-resuscitative gasps^34,35,52^. During gasping, breaths become punctuated large amplitude events with long inter-gasp intervals, larger diaphragmatic activity and recruitment of more auxiliary inspiratory muscles (Fig. 7a)^35^. Inspiratory, expiratory and tonic neurons of all genotypes have reduced firing rates on average (Fig. 7b–d and Extended Data Fig. 10a–c). Activity during the inter-burst interval is reduced across the population as evidenced by an increase in respiratory tuning strength (Extended Data Fig. 10f). Many normally expiratory neurons reconfigure and become active during inspiration (Extended Data Fig. 10d,e).

Importantly, the rotational dynamics observed during eupnea are diminished or lost during gasping. Instead, the low-dimensional trajectories reconfigure to exhibit all-or-none, ballistic dynamics in which both pre-inspiratory and post-inspiratory activity is lost, and the PCs evolve only during the gasp effort. (Fig. 7e–g). During gasping, the population is constrained to a stable region between gasp efforts, and each gasp is characterized by a quick and symmetric excursion away from, and back to, this region (Fig. 7e–g and Supplementary Video 6). Further, the transition from eupnea into gasping is smooth and characterized by a progressive ‘shrinking’ of the rotational trajectories (Extended Data Fig. 10i). To examine the structure of the latent dynamics during gasping, we refit the rSLDS model to the period of time in which the animal was gasping, and only for the recordings that were regenerative during normal breathing (*n* = 11). We find that the eigenvalues no longer cleanly separate into distinct dynamics for inspiration and expiration as they did during eupnea (Fig. 7h and Supplementary Fig. 5). Lastly, we quantify the changes in correlation structure during gasping as was done for morphine and sighs (Fig. 7i–k). The correlation structure is drastically altered during gasping across all recordings, indicative of a VRC wide reconfiguration.

## Discussion

Growing adoption of large-scale population recording and analysis techniques has driven widespread appreciation of low-dimensional patterns as a ubiquitous feature of neural circuits^49,53^. Here we show that this framework elucidates the network dynamics of distributed VRC populations that are critical to generating, patterning and maintaining breathing. The VRC population evolves through rotational trajectories on a low-dimensional manifold of neural activity space that are consistent across individual animals. These trajectories target an ‘inspiratory-off’ attractive region, expanding the well-described phenomenon of an inspiratory off-switch^25–27^ to the network activity of the entire VRC. Further, VRC populations can be well simulated by a simple dynamical model in which inspiration and expiration, respectively, are governed by alternation between discrete, intersecting linear dynamical regimes. The piecewise linear model used here does not necessarily imply that the biological system performs a discrete switch between linear systems, but that the underlying nonlinear dynamics that govern the system are well approximated by these linear dynamics. Practically, quantitative comparisons across animals and conditions are afforded by the analysis of the eigenvalues of the constituent linear systems.

The high-resolution mapping data support the general organizational structure of the VRC as proposed in previous models^6,7^, but also reveal that these models are underresolved to fully capture the distributed, heterogenous nature of the neural populations that underlie breathing and its associated behaviors. Inspiratory neurons are more numerous, exhibit higher firing rates and are more coherent than expiratory neurons, potentially contributing to their over representation in local field potential analyses, and their historic preoccupation in the literature^9^. However, we observe a critical importance of the expiratory phase in the population dynamics, particularly as a low-dimensional temporal reorganizing feature for the entire population. The combined anatomical, transcriptional and electrophysiological characterization here highlights the potential for overlapping functional populations. Optogenetic and/or chemogenetic manipulations of subsets of neurons based on anatomical and transcriptional factors may still target intrinsically heterogeneous populations that could be performing orthogonal functions. We note that light scattering causes optogenetic tagging efficiency to decreases rostrally (Supplementary Fig. 2). Neurons cannot be identified by a lack of optical response, which thus limits the completeness of cell-type characterizations. However, we expect this drop in tagging efficiency should affect all driver lines equally, and we still observe differential distributions of tagged neurons even rostrally (for example, a small population of SST^+^ expiratory neurons are found predominately rostrally). Our data further support the idea that discrete boundaries between VRC subdivisions are as not apparent in freely breathing animals^9,54^. We emphasize that these conclusions do not contradict the fact that the preBötC is necessary for breathing when lesioned acutely^34,55^ or when isolated in slices^12^, nor that the BötC is a region within the VRC that is biased towards inhibition of inspiration^6^.

This work is a significant advance on previous efforts to create maps of VRC activity patterns. Recently, Dhingra et al.^56^ performed a comprehensive mapping of local field potentials across the isolated rat brainstem. A large body of work from Morris, Lindsay and colleagues^46,57^ also recorded from many isolated respiratory units simultaneously in cats. The data presented here are restricted to the ventrolateral medulla but offer advances on those works in that we record simultaneously from an order of magnitude more single units in animals with a fully intact neuro-respiratory system breathing in a dynamic regime similar to that during awake, quiescent breathing. Importantly, we find that the proliferation of activity-based cell classes are better quantitatively described as a continuum of activity patterns rather than sets of discrete types, at least when considered on the basis of respiratory-related electrophysiological activity patterns (Fig. 2). Further, we employ optogenetic tagging to identify and characterize neurons that express particular transcriptional markers (for example, excitatory versus inhibitory) and detail their anatomical and electrophysiological characteristics.

Analyses of latent population dynamics allow for quantitative and qualitative investigation of the activity patterns of large populations of respiratory neurons. We find two salient features of the latent trajectories that are consistent in both the PCA and rSLDS models. First, the trajectories evolve continuously throughout the respiratory phase, and second, inspiration-off/expiration-on represents a highly attractive region in the latent space. Importantly, trajectories evolve continuously during the expiratory period in which there is no overt diaphragm activation. This is corroborated by the single neuron phase tuning curves that continuously tile the respiratory phase. This continuous representation is lost during gasping and is thus probably not strictly necessary for rhythmic inspiratory efforts. We speculate that these continuous dynamics may be supported through inputs from vagal afferents and inhibitory populations that permit fast respiratory rates.

Low-dimensional analysis of respiratory populations revealed that the respiratory networks evolve through a rotational trajectory. This is particularly important for breathing, which involves the temporal coordination of numerous muscles (including abdominal, laryngeal and oropharyngeal) that must be differentially activated and inactivated to generate a functional breath^58^. We hypothesize that a rotational trajectory creates a temporal framework upon which motor effectors can be sequentially and continuously timed. This may also allow other orofacial behaviors to ‘read out’ the appropriate respiratory phase from the ventrolateral medullary population. Within the rotational dynamics, the inspiratory-off attractor is a crucial feature that may allow for precise timing of these behaviors within the respiratory phase. For example, vocalization, swallowing and coughing all occur predominantly during the expiratory phase^59,60^. We further support this notion with our observation of a preponderance of inhibitory, expiratory neurons in the intermediate reticular nucleus, a region that hosts circuits for a multitude of orofacial behaviors^21,22,59^. An open question remains whether activation of phase-delaying behaviors such as vocalization evokes an excursion within the manifold which effectively increases the radius of the trajectory (akin to the observations of sighs during the inspiratory phase), or if these behaviors would evoke an ‘off-manifold’ excursion into an orthogonal region of the latent space.

A major advantage afforded by large-scale recording and subsequent low-dimensional analyses is that it allows intuitive interpretation of network-wide compensations to perturbations that would otherwise be impenetrable. Systemic opioid administration evokes widespread and diverse changes in individual cell spiking activity. Optogenetic tagging and large-scale recordings together suggest a potentially important effect of increases in the activity of tonic inhibitory neurons during opioid administration. When taken alone, the changes of single units ‘in a vacuum’ in response to opioids underresolve the covariation that is present in the population. Importantly, the low-dimensional rotational behavior of the VRC is largely preserved during OIRD, suggesting the possibility of compensatory network solutions to maintain robust breathing. The overarching effect of OIRD can be described as a slowing of the network dynamics. Opioids have been demonstrated to cause both hyperpolarization of Oprm1^+^ neurons as well as decreases in synaptic efficiency^30^. These mechanisms may, at the level of the network dynamics, impose a ‘frictional effect’, which in the extreme may sufficiently slow breathing to result in inescapable hypoxia.

Subsequently, hypoxia-induced gasping results in a collapse of the rotational dynamics. This collapse implies that network mechanisms that support normal breathing are no longer maintained. Given that the neural population collapse into gasping is continuous, it may be the case that there is not a discrete ‘switch’ or triggering mechanism involved in evoking gasping. Instead, gasping may come about as a degeneration of the normal eupneic behavior as it gradually becomes unable to be sustained by network interactions. These data augment observations in slices that show the cellular and molecular mechanisms governing rhythmic activity during eupnea are fundamentally different than that of gasping^52^; the latter is selectively dependent on serotonergic and noradrenergic mechanisms^61–64^. These modulatory systems have been found to be disrupted in children that died of SUIDS^36–38^. Importantly, the serotonergic disturbances are widely distributed in the medulla and include the nucleus gigantocellularis located in the rostral medulla, an area that is critical for arousal^65^. We speculate that these disturbances remain undetectable under normal breathing conditions because the VRC-wide rotational coordination between inspiratory and expiratory populations imbues the respiratory network with increased robustness. However, during hypoxic and gasping bouts, when the network dynamics transition from rotational to ballistic dynamics along the entire VRC, these rostrally located disturbances may become fatal in children susceptible to SUIDS.

The large-scale neural population dynamics associated with behaviors so stereotyped as breathing are just beginning to be explored^4,66^. The consistency of low-dimensional structures across animals seen here, and the similarity to the neural rotations in the spinal cord described by Lindén et al.^4^ suggest that stereotypical behaviors may be governed by fundamental neural dynamics that are similar across motor behaviors and species. The comparable low-dimensional structure of neuronal population dynamics contrasts with the striking cellular diversity and drastic differences in the biological substrate responsible for the generation and reconfiguration of these rhythmic behaviors. This suggests the possibility of a convergent and/or degenerate solutions for neural systems to perform computationally similar operations in disparate systems^5^.

## Methods

### Animals

All procedures were approved by the Seattle Children’s Research Institute Institutional Animal Care and Use Committee. Both male and female mice aged p46-p155 were used. Homozygous Vglut2^Cre^ (Jax# 028863), Vgat^Cre^ (Jax# 016962), ChAT^Cre^ (Jax# 031661), SST^Cre^ (Jax# 013044) and Dbx1^CreERT2^ (donated by Dr. Del Negro College of William and Mary, VA) mice were crossed with homozygous Ai32 mice (Jax# 012569) that contain a flox-STOP-flox sequence fused to channelrhodopsin (ChR2) and enhanced yellow fluorescent protein at the Rosa26 locus. Thus, offspring expressed ChR2 and enhanced yellow fluorescent protein only in the neurons that expressed the promoter for the Cre driver. In Dbx1^CreERT2^ animals, tamoxifen (24 mg kg^−1^ intraperitoneally) was injected at E10.5 to target neurons of the preBötC^42^. Animals were randomly selected for recording based on availability. Data collection and analysis were not performed blind to the conditions of the experiments.

### In vivo surgical preparation and data acquisition

Anesthesia was induced in 3% isoflurane and urethane (150 g kg^−1^) was administered intraperitoneally; animals were then removed from isoflurane. Animals were placed on a temperature regulating heating bed (Kent Scientific) and maintained at 37 °C for the remainder of the experiment. Two fine (0.005 inch diameter) stainless steel wire EMGs (AM-systems) were inserted into the right diaphragm. The EMG was amplified (10,000×) and filtered (100 Hz to 5 kHz) (AM-systems 1700) before being digitized at 10 kHz (NI PXIe-8381). Ear bars were used to affix the head in a stereotactic frame. The skin overlying the scalp and neck musculature was removed and the skull surface cleared (Supplementary Fig. 1). The neck musculature was removed to expose the occipital bone, dural surface just ventral to the cerebellum, and the first cervical vertebra. The skull was then leveled such that bregma and lambda lie in the horizontal plane. A titanium headplate was then affixed to the skull surface with ultraviolet cure dental cement. The animal was removed from the stereotactic frame and repositioned in a separate headfixing frame equipped with a motorized micromanipulator. A custom 3D-printed nosecone was placed on the nose of the animal, and 100% O_2_ was then supplied to the mouse. We carefully removed the dura overlying the left caudal surface of the brainstem with a #11 scalpel, taking care not to damage underlying brain tissue or vasculature. A single Neuropixel probe (IMEC) was dipped in DiI (Thermofisher V22885) and then placed in a motorized manipulator (Sutter MPC-325) positioned caudal to the animal. The probe was aligned in the horizontal plane, with the probe tip toward the animal. We then find what we term the ‘skull apex’ point (Supplementary Fig. 1), the point at the midline of the suture between the intraparietal and the occipital bone. We then target the VRC at 1.25 mm lateral, 4.8 mm ventral to this point. The probe is advanced to touch the caudal surface of the brainstem, then advanced 4 mm into the brainstem at a rate of approximately 1 mm min^−1^. The probe settles for ≥15 min before recording begins. Neural and EMG data are acquired with SpikeGLX. Gas presentation was controlled by electrical solenoid valves (Beduan) connected to a single flowmeter (Cole Parmer). Timing of solenoid valves and laser pulses (see ‘Optical tagging’ section) were controlled by a Teensy 3.2 microcontroller. In some recordings, a single dose of morphine (150 mg kg^−1^) was administered intraperitoneally following a baseline recording period (3–5 min) and the optical tagging protocol. After recording, the probe is retracted, and repositioned approximately 100 μm away in either the mediolateral or dorsoventral axis before another insertion and recording procedure. Following all recordings, the probe is extracted and placed in 1% Tergazyme (Sigma-Aldrich Z273287) overnight. The animal is perfused with saline and 4% paraformaldehyde (PFA), and the brain is removed for histological processing.

### Optical tagging

During recording, a Cobalt 473-nm laser is attached to a 600-μm-diameter 0.22 numerical aperture fiber optic with a cleaved end (Doric). Fiber power measured at the tip was 50–70 mW; high power and large fiber diameter was used to increase the likelihood of evoking spiking in rostrally located neurons that are far from the caudal surface of the brainstem. The fiber optic was placed just caudal to the brainstem surface. To perform optical tagging, 75 sequential 10-ms laser pulses with a 1.5-ms-duration sigmoidal on-ramp and off-ramp and a 3-s delay between each pulse was presented to the caudal surface of the brainstem (Supplementary Fig. 1). This causes light-evoked spiking in neurons that express ChR2. This stimulation can directly alter respiratory activity and so periods of opto-tagging are not used in analyses. Positively tagged neurons are determined using the stimulus-associated latency test^24^. Neurons with a stimulus-associated latency test *P* < 0.0001 and spiking on at least 25% of stimulations were considered positively tagged.

### Data processing and spike sorting

The action potential band (acquired at 30 kHz) Neuropixels data were filtered (CatGT global demux), and spikes were extracted, drift corrected and sorted using Kilosort3 (for KS3 parameters, see Supplementary Table 1). Putative single units were then quality sorted using the ecephys pipeline^67^. Double-counted spikes were removed. Only units that were classified by the ecephys pipeline as not ‘noise’ units had inter-spike interval violations <0.5, amplitude cutoff <0.1 and a presence ratio >0.9, and were also labeled as ‘good’ by KS3 were retained for analysis. This restrictive filtering potentially missed viable single units, but probably rejected most false positive units^67^. Administration of morphine caused complete silencing and recruitment of units. Thus, for analysis of recordings with morphine administration the presence ratio threshold was relaxed to 0.25. Units from these recordings are included only in Fig. 5 and Extended Data Figs. 7–9 as they apply to OIRD.

Raw diaphragm EMG data (acquired at 10 kHz) were processed offline using custom software. First, the electrocardiogram signal was removed, the signal was bandpass filtered between 300 and 5,000 Hz and rectified, a 50-ms median filter was applied, and the signal was downsampled to 1 kHz. The amplitude of the diaphragm signal was then normalized by dividing by the s.d. Diaphragm bursts were detected using the scipy ‘find_peaks’ function. Units were identified as putatively axonal and somatic on the basis of the method described in Sibille et al.^41^. Waveshape features were calculated as in Sibille et al., whitened and projected onto the leading two PCs. A manual decision boundary was drawn to separate the two densities in that space (Supplementary Fig. 2c). Some units were labeled as neither axonal nor somatic.

### Probe localization

Before inserting the probes into the caudal brainstem, they were dipped five times, for 5 s each dip, in DiI (Thermofisher V22885), a red fluorescent dye, to mark the probe track. After the final recording of the procedure, the animal was euthanized, perfused with phosphate-buffered saline, followed by 4% PFA, and the brain is removed. The brain was fixed overnight in 4% PFA, 15% sucrose for 24 h and finally 30% sucrose. The brain was embedded in embedding medium (NEG-50) and frozen at −80 °C. Then 25-μm coronal sections were collected, and imaged on an Olympus BX61VS slide scanner at 4× magnification using Olypmia VS-ASW. We then used SHARP-Track^68^ to downsample and manually transform each image to match a corresponding section of the Allen CCF. The fluorescent probe tracks were identified in the registered slices and reconstructed in 3D atlas space. Special care was taken to accurately identify the probe tip position (that is, the most rostral slice that DiI staining could be observed) to accurately reconstruct the rostrocaudal positioning of the probe, and the respective channels. We then visualized the activity of the probe alongside the extracted anatomical location to fine tune the rostrocaudal position of the probe. Units that projected below the ventral surface of the Allen CCF were omitted from analyses.

### Anatomical subdivision of VRC and medulla

Since the preBötC and BötC are not defined in the AllenCCF, we create a manual subdivision of the paragigantocellular reticular nucleus, lateral part (PGRNl) to create these regions in our analyses. We define BötC as extending from the caudal edge of the VII (−5.96 mm AP) for about 400 μm in the caudal direction (−6.4 mm AP); preBötC as extending from this caudal edge (−6.4 mm AP) to include the remaining PGRNl (−6.85 mm AP). We restrict both preBötC and BötC such that the medial edge is in line with VII (0.89 mm lateral). Any PGRNl units that do not fall into these defined regions remained labeled as PGRNl. Lastly, we combine the superior olivary complex lateral (SOCl) and medial (SOCm) parts into one region (SOC).

### Coherence calculations

Coherence estimates whether two signals (here the breathing activity and a given neuron’s spiking activity) share frequency components. We use Chronux^69^ to compute the multi-taper coherence of the continuous integrated diaphragm signal (subsampled to 1 kHz) with the point process signal of the spike times for a given unit within the first 5 min of a recording (coherencysegcpt). We use a time bandwidth product of 3 with 5 tapers. We compute the lower and upper bound on coherence using Jackknife error method with error bounds of [0.001, 0.999]. Chronux computes the phase lag of the unit (*ϕ*) at each frequency component. We determine the phase lag of a unit to be the value of *ϕ* at the frequency that is maximally coherent between the diaphragmatic signal and the unit’s spiking activity.

In addition to computing coherence, we also compute the directional selectivity index *L* (ref. ^70^) of each unit by:

$$L=\left|\frac{{\sum }_{j}r({\phi }_{j})\exp (i{\phi }_{j})}{{\sum }_{j}r({\phi }_{j})}\right|$$

where *r*(*ϕ*) is the phasic firing rate of the unit as a function of respiratory phase, and *ϕ*_*j*_ is the value of phase in the *j*th phase bin. *L* is 1 − *σ*^2^ where *σ*^2^ is the circular variance of the firing rate as a function of respiratory phase.

### Phase computation and phasic responses

Phase (*ϕ*) is defined here from −*π* to *π* to normalize breaths across time and animals. We set the onset of each breath to *ϕ* = 0 and offset of each breath to *ϕ* = *π*. We then linearly interpolate a value of *ϕ* for each time sample between onset and offset. We set the sample immediately following breath offset to *ϕ* = −*π*, and linearly interpolate the samples until the next breath onset on the interval [−*π*, 0). Phasic responses of individual neurons is computed as $\frac{P\left(\phi |{{\mathrm{spike}}}\right)}{P(\phi )}$. Note, the phase lag of a unit (defined in ‘Coherence calculations’ section) is not necessarily the phase of the respiratory cycle for which that unit’s spike rate is maximal.

### Activity-based cell class decomposition

We perform unsupervised clustering to identify classes of neurons on the basis of their phasic activity patterns. We first compute the phasic activity of a given unit as above (‘Phase computation and phasic responses’ section) to get firing rate as a function of breathing phase. We bin the respiratory phase into 100 bins ($\frac{\pi }{50}$ radian bin width). We then smooth the phasic curve with a three-sample Savitzky–Golay filter and normalize to that unit’s maximal firing rate. To account for possible dimensionality issues when clustering the phasic data, we create three sets of data to be clustered independently. The first set is the raw firing patterns, which is a matrix *R* of size *n* by *t* where *n* is the number of units and *t* is the number of phase bins (100). We create the second set by performing PCA decomposition on the matrix *R*. We retain only the leading four PCs, which account for >95% of the variance of *R* to create *P*, which is of size *n* by 4. We create the third set *N* by performing nonnegative matrix factorization decomposition on *R*. We retain 20 nonnegative matrix factorization components, such that *N* is of size *n* by 20. We then cluster each set of data using KMeans clustering and Ward agglomerative clustering (sklearn). We vary the desired number of clusters from 2 to 15. We then compute the silhouette score for each combination of data preconditioning, clustering method and number of desired clusters. Controls are performed by randomly shuffling the cluster label assigned to each unit and recomputing the silhouette score. Shuffles are repeated 5,000 times, and the 99th percentile silhouette score is reported. Lastly, we repeat this analysis for subsets of *R* that include only units that have a lower bound of coherence value greater than a given threshold *T* where *T* = [0, 0.1, 0.3, 0.5, 0.7, 0.9, 0.99].

### Low-dimensional population analyses

To compute the PCA decompositions of the populations, we first convert the spike times into a normalized, smooth spike rate by binning the spike rates into 5-ms bins, smooth the bins with a Gaussian kernel with *σ* = 10 ms and compute the square root transform. The smoothed spike rates form a matrix *X* of size ‘number of neurons by number of time bins’, which is passed to the PCA. We fit PCA on data obtained during 100% O_2_ presentation and apply that PCA decomposition to the entire recording.

For Extended Data Fig. 8 we compute the PCA decompositions for a given recording separately on each condition, which results in two square matrices *A*, *B* each of size *n* by *n* where *n* is the number of units. We compute the principal angles^71^ by first computing the singular value decomposition (SVD) of the product of these two matrices:

$$U\varSigma {V}^{T}={{\mathrm{SVD}}}({A}^{T}B)$$

where *Σ* is a diagonal matrix in which the diagonal entries are the cosines of the principal angles *θ* between the subspaces, ranked by decreasing magnitude. We report the cos(*θ*) as ‘similarity’.

In addition to PCA, we also fit rSLDS^48^. For detailed examples and explanations, see https://github.com/lindermanlab/ssm. Briefly, these models consist of *K* linear dynamical systems of the form:

$${\bf{x}_{t}}=A{\bf{x}_{t-1}}+\bf{b}+\it{{w}_{t}}$$

where **x**_*t*_ is the low-dimensional continuous state vector of the system at time *t*, *A* is the matrix that specifies the continuous state update at each time step, **b** is a bias vector and *w* is a noise term. **x**_*t*_ and **b** have dimension *D*, and *A* is a [*D* × *D*] matrix. In the rSLDS, there are *K* number of *A* matrices and **b** vectors, one for each discrete state *k*, which we refer to as *A*_*k*_ and **b**_*k*_. When the system is in discrete state *k*, the continuous state is now updated as:

$${\bf{x}_{t}}={A}_{k}{\bf{x}_{t-1}}+{\bf{b}_{k}}+{{w}_{k,t}}$$

In comparison with a switching linear dynamical system (SLDS) in which the transition between the discrete states (*k*) is dependent only on the previous discrete state, switches between states in the rSLDS are dependent on the value of the continuous state **x**_*t*_. In other words, the low-dimensional representation of neural activity will affect the probability of being in a discrete state. More precisely, the probability of the current discrete state *z*_*t*_ transitioning from discrete state *j* to discrete state *i* is given by:

$$p\left({z}_{t}=i|{z}_{t-1}=j,{\bf{x}_{t-1}}\right)\propto \exp \left(\log \left({\bf{R}_{i}^{T}}{\bf{x}_{t-1}}\right)\right)$$

**R**_*i*_ is a vector that weights the previous state $\bf{x}_{t-1}$.

We fit the rSLDS models with the number of discrete states *K* = 2 and the dimensionality of the continuous state *D* = 2. We use an isotropic Gaussian noise model for the dynamics. We employ a Poisson emissions model so that the mapping between the continuous state **x**_*t*_ and the estimated neural activity **y**_*t*_ (where **y**_*t*_ is a vector of spike counts of length # neurons at time bin *t*) is a GLM with a Poisson distribution. We pass the spike counts in 10-ms bins for all neurons during 100% O_2_ presentation as the inputs to fit the rSLDS model.

This fits two matrices *A* and bias vectors **b**—one for each of the *K* states—as well as defines the transition dynamics between these two dynamical systems. *A* and **b** define the flow fields in Fig. 4. We then supply an initial ${\widetilde{\bf{x}}}_{0}$ to generate simulated continuous states forward in time ${\widetilde{\bf{x}}}_{t}$ that depend only on the fit dynamics. We set the noise term *w* to zero after the observation that including a *w* term resulted in simulated states that were far more variable than the observed states, and that states simulated with omission of the *w* resembled the observed states. Lastly, we perform support vector regression (scikit-learn) to generate a mapping from the observed states *x*_*t*_ to the observed integrated diaphragm activity. We then apply that mapping to the simulated continuous states ${\widetilde{\bf{x}}}_{t}$ to generate a simulated diaphragm activity. The generative models either evoked a simulated diaphragm activity that exhibited periodicity and resembled normal diaphragm activity or did not generate periodic activity (nonregenerative). This distinction was clear, and no qualitatively in-between types of simulated diaphragm activity was observed. We tested whether a dynamical system with *K* = 2 discrete states was necessary and sufficient to result in a generative model capable of simulating diaphragm activity by fitting models with *K* = 1 and *K* = 3, respectively (Extended Data Fig. 5).

To test the robustness of the regenerative models and the relative importance of highly coherent and high firing rate neurons we perform a dropout analysis of the rSLDS model. For each recording that was capable of generating simulated diaphragmatic activity (regenerative), we sequentially removed neurons in order of their coherence (both increasing and decreasing), firing rate (highest firing rates first) or randomly, and then refit the rSLDS model and the subsequent support vector regression to the observed diaphragm. We then compute how many neurons can be removed from the population while retaining simulated diaphragmatic activity. If the dropout model’s simulated diaphragm activity exhibited a frequency 75–150% and inspiratory duration 75–125% of the full model’s simulated diaphragm activity it was considered regenerative. We fit a logistic regression on the binary variable of regenerative or not as a function of number of units dropped and determine the decision boundary of that logistic regression. In cases where very few units could be dropped while maintaining regeneration, the decision boundary was below one unit dropped despite maintenance of regeneration; we therefore define the number of units to failure to be either the decision boundary or the first failed regeneration, whichever is greater. Lastly, we divide by the total number of recorded neurons to report the percent of the population that can be lost while maintaining diaphragm activity that resembles the full model.

To align the latent dynamics of the rSLDS models, we first compute the average of the 2D continuous state **x**_*t*_ as a function of phase by computing $\frac{P\left({\phi }_{i}|\bf{x}\right)}{P({\phi }_{i})}$ where *ϕ* is subdivided into 100 bins of size $\frac{\pi }{50}$ radians. This gives us a (100 by 2) matrix for each recording. We choose one recording to be the ‘target’ recording, which we denote as *Z* and all others as source recordings, which we denote as *X*. We then compute the 2-by-2 alignment matrix *W* as:

$$W={X}^{-1}Z$$

We then compute the aligned state: ${\hat{\bf{x}}}_{t}={\bf{x}}_{t}W$.

### Sigh and gasp detection

Sighs were detected by first computing the area under the curve of the integrated diaphragm signal for each breath. We then compute the rolling median absolute deviance (MAD) with a centered 51 breath window. This gave, for each breath, the MAD of the 25 previous and 25 following breaths. Breaths for which the area under the curve of the integrated diaphragm was greater than seven times the surrounding MAD were identified as sighs.

Periods of gasping were detected only during hypoxia presentation by first computing the IBI for each breath. We smooth the IBI with a centered median filter with window length of seven breaths. If the smoothed IBI exceeded 1 s, a gasping period is determined to begin. The gasping period was deemed concluded if the smoothed IBI became shorter than 0.85 s.

### Statistics

Sample sizes were not determined a priori with a power analysis. Individual datapoints are shown where feasible, and nonparametric tests are used where data are expected to be nonnormal. Otherwise data were assumed to be normal, but this was not formally tested. No animals were excluded from data analysis. In some cases, mice failed to resuscitate from hypoxia exposure; such recordings were not used.

### Reporting summary

Further information on research design is available in the Nature Portfolio Reporting Summary linked to this article.

## Online content

Any methods, additional references, Nature Portfolio reporting summaries, source data, extended data, supplementary information, acknowledgements, peer review information; details of author contributions and competing interests; and statements of data and code availability are available at 10.1038/s41593-023-01520-3.

### Supplementary information

Supplementary Information Supplementary Figs. 1–5, Video Legends 1–6, references and Table 1.

Reporting Summary

Supplementary Video 1 3D reconstruction of all probe insertions, colored by mouse genotype. Colors as in Fig. 1.

Supplementary Video 2 Anatomical locations of all recorded neurons. The outline of the entire brain as well as the VII and nucleus ambiguus are shown. Gray neurons on the left side are tonic neurons, light-purple neurons on the right side are weakly phasic neurons (0.1 < coherence lower bound < 0.9) and dark-purple neurons on the right side are strongly phasic neurons (coherence lower bound >0.9). Note that the strongly phasic neurons are found in a more restricted region of the ventrolateral medulla than either the tonic or weakly phasic neurons and are not often found within VII. Visualization performed with Brainrender.

Supplementary Video 3 Neural population activity evolves through constrained trajectories in a low-dimensional space. Integrated diaphragmatic activity (top) and activity of the 249 simultaneously recorded neurons (middle) in Fig. 2 evolve over time. Firing rates are binned at 5 ms and smoothed with a Gaussian kernel of s.d. 15 ms. Firing rates are normalized to maximum firing rate for visualization. Purple is 0 firing rate; yellow is maximal. Trajectories evolve over time through constrained PC space (bottom). Color of moving trace in bottom indicates diaphragmatic activity (black is inactive; yellow is maximally active). Video speeds from 15% real time to 50% real time after several seconds of playback. The inspiration-off attractor described in Fig. 2 is appreciated in the temporal evolution of the neural population trajectories. A sigh and associated post-sigh eupnea is observed early in the video; the neural population trajectory exhibits an excursion from the typical eupnea trajectory.

Supplementary Video 4 Regions of a stable neural manifold correlate to multiple respiratory features. Each quadrant of the video shows the position of the neural population in the space defined by the leading three PCs for a 1,000-s time period, with each dot representing a single 5-ms time bin. Quadrants are colored by the diaphragmatic activity (top left), trajectory speed (top right), fourth PC (bottom left) and respiratory phase (bottom right).

Supplementary Video 5 Morphine slows neural population trajectories through PC space. Red line shows trajectory evolution at 30% real-time speed in control (left) and morphine (right). Colored dots show 50 s of overlapping neural states, where each dot is a 5-ms bin. Dots are colored by trajectory speed.

Supplementary Video 6 Transition from eupnea to gasping and recovery. Video laid out as in Supplementary Video 3. Ninety-seven neurons are shown in the raster. Video plays at 100% real time of recording. Rotational trajectories through the PC space are observed during eupnea. These rotations become ballistic efforts during gasping (brown traces). During recovery, the trajectories (blue) smoothly transition back to normal eupneic rotations.

### Source data

Source Data Fig. 1 Statistical source data.

Source Data Fig. 2 Statistical source data.

Source Data Fig. 3 Statistical source data.

Source Data Fig. 4 Statistical source data.

Source Data Fig. 5 Statistical source data.

Source Data Fig. 6 Statistical source data.

Source Data Fig. 7 Statistical source data.

## Data Availability

Data presented in this paper are available upon reasonable request. Source data are provided with this paper.

## Code availability

Custom MATLAB and Python code was used to process and visualize the data in this study. All codes are available upon reasonable request.
