# Supplementary material for: Latent neural population dynamics underlying breathing, opioid-induced respiratory depression and gasping
Source: Nat Neurosci. 2024 Jan 5;27(2):259–71. doi: 10.1038/s41593-023-01520-3 (PMC10849970; doi:10.1038/s41593-023-01520-3)
Supplement: Supplementary file 1 — Supplementary Figs. 1–5, Video Legends 1–6, references and Table 1. [file 41593_2023_1520_MOESM1_ESM.pdf]

nature neuroscience

Article https://doi.org/10.1038/s41593-023-01520-3

Latent neural population dynamics underlying breathing, opioid-induced respiratory depression and gasping

In the format provided by the authors and unedited

Supplementary Figures

Supplementary Figure 1

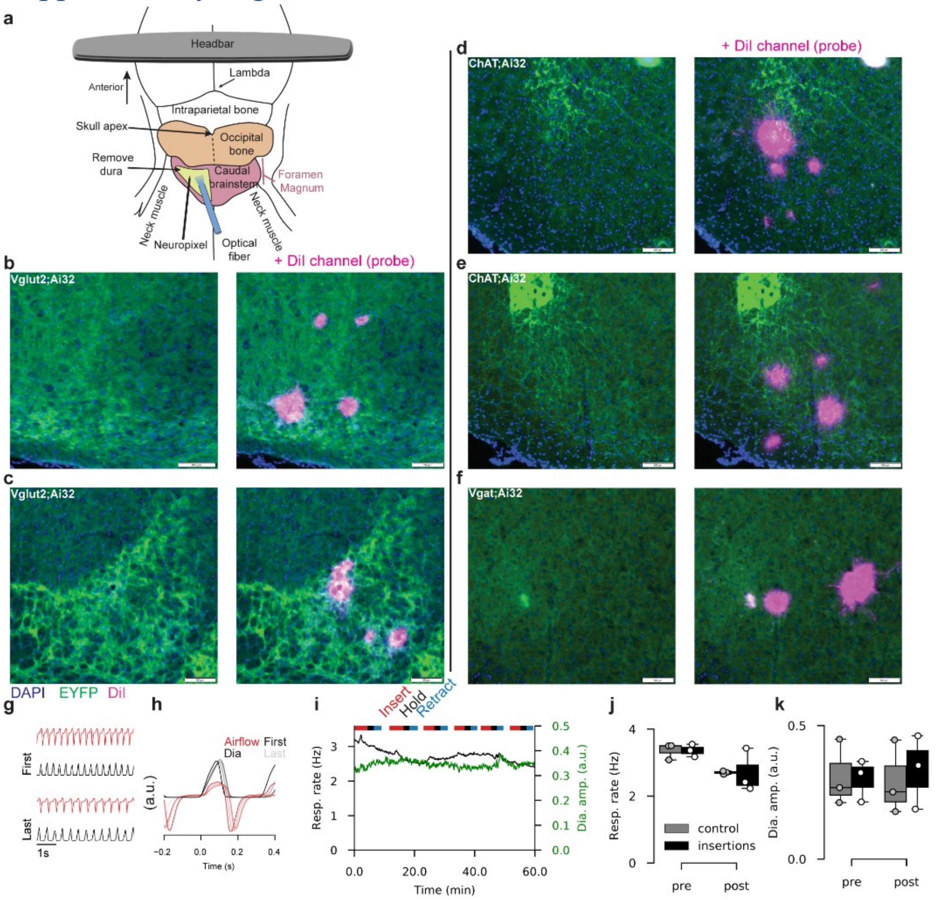

Surgical preparation causes minimal damage to VRC and alterations to normal respiratory behavior (**a**) Schematic of surgical preparation. (**b-f**) Five example coronal sections from fives mice without (left) and with (right) the DiI channel visible. Histology from all 36 mice was analyzed and minimal damage was observed in all cases. Blue is DAPI, green is Cre-driven EYFP, magenta is DiI tract indicating probe track. Probe penetrated in the anterior-posterior direction (perpendicular to the image plane). Smaller radii tracts indicate penetrations later in the experiment in which DiI has dissipated. (**g-k**) We perform separate tests of the effect of repeated penetrations of the VRC on breathing. In 3 urethane anesthetized mice, we perform 6 sequential insertions per mouse of the VRC spaced in a 100-150μm grid. Probes were inserted over ~5 minutes, held stationary for 3 minutes, and retracted for ~3 minutes. Nasal airflow and diaphragm activity were monitored. In 3 control mice we perform the same surgical preparation including durotomy but perform no probe insertions. (**g**) Example airflow (red) and integrated diaphragm (black) after the first (top) and last (bottom) insertion of the probe. (**h**) Breath aligned average airflow (red) and integrated diaphragm (dia., black) after each penetration. Later recordings are shown in increasing transparency. No qualitative differences are observed in respiratory behavior. (**i**) Respiratory rate (black) and diaphragm amplitude (green) over the six insertions in an example mouse. Lines at top

of trace indicate insertion, holding, and retraction periods. Reduction in respiratory rate is observed, but no change in diaphragmatic amplitude is observed. (**j**) Respiratory rate and (**k**) diaphragmatic amplitude before and after all insertions (black boxes: mean+/- IQR; white dots are individual mice, n=3 mice). Mice in which no insertions were performed are compared at 90 minutes after initiation of recording (gray boxes: mean +/-IQR; gray dots are individual mice). There are transient increases in respiratory rate during insertion periods. Respiratory rate depression in mice without VRC insertions exhibit similar respiratory depression over time, likely due to prolonged urethane anesthesia. Thus, repeated insertion of the probe likely does not cause significant disruption of the VRC respiratory networks.

Supplementary Figure 2

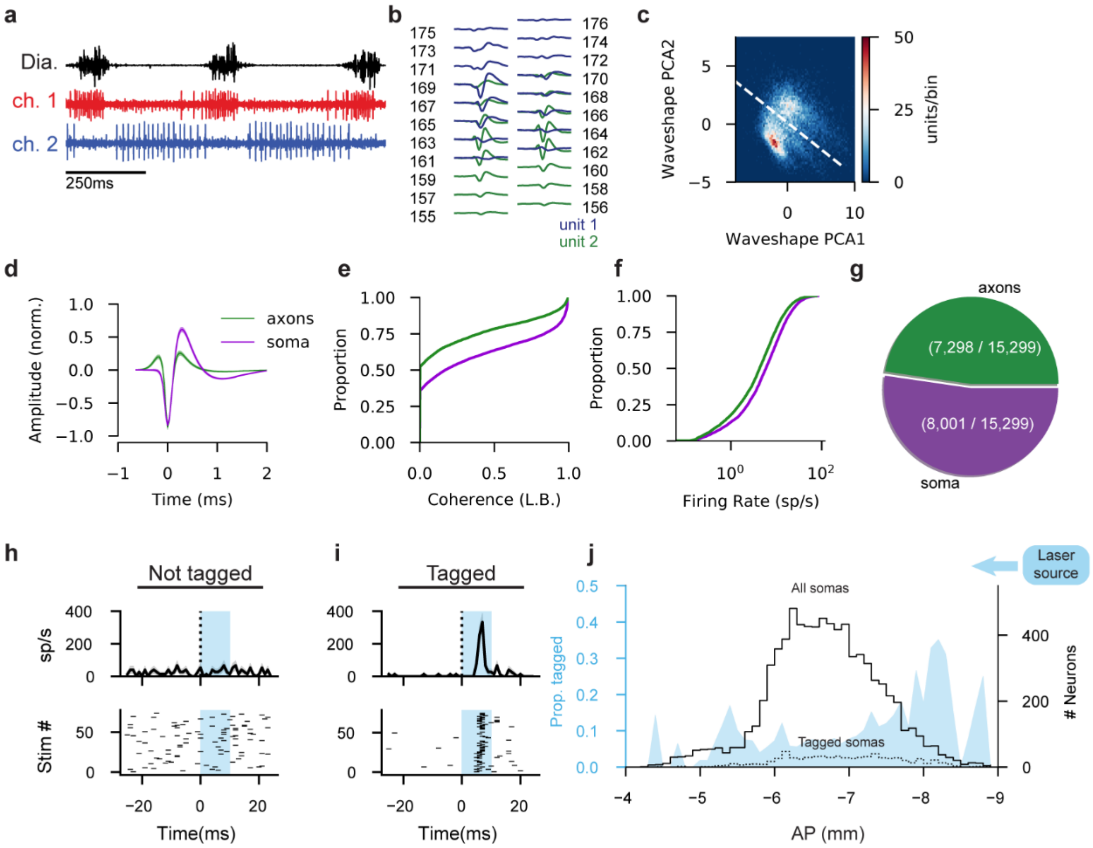

(**a**) Example raw signal from the diaphragm and two example channels with clear inspiratory and expiratory (red and blue, respectively) single units. (**b**) Average waveforms of two separable single units recorded simultaneously on overlapping electrode channels. Channel number is indicated. (**c**) PCA projections of waveshape features as detailed in Sibille et al.[1]. White dashed line is manual decision boundary separating putative axonal units from putative somatic units (**d**) Average waveform shape of putative axons (green) and soma (purple). Shaded regions are mean +/- 10*S.E.M. (**e**) Cumulative proportion of lower bound on coherence and (**e**) firing rate of axonal vs. somatic units. Somatic units are more likely to be strongly respiratory and have higher firing rates, suggesting the separation of axonal and somatic units is not arbitrary. (**g**) Proportion of axonal vs. somatic units of all single units obtained. (**h,i**) Optogenetic tagging results for an

untagged neuron (h) and a tagged neuron (i). Shaded regions are presentation of a 10ms 473nm laser pulse with a sigmoidal on- and off ramp. Top: Stimulus aligned average firing rate ±S.E.M. Bottom: Stimulus aligned spiking for each of 75 laser pulses, each row is a stimulation, each bar is a spike. (**j**) Anterior-posterior distribution of positively tagged and untagged cells. Shaded region is proportion tagged (left abscissa), lines are absolute number of neurons (right abscissa) across all animals and genotypes. Laser is positioned posteriorly (right of histogram).

Supplementary Figure 3

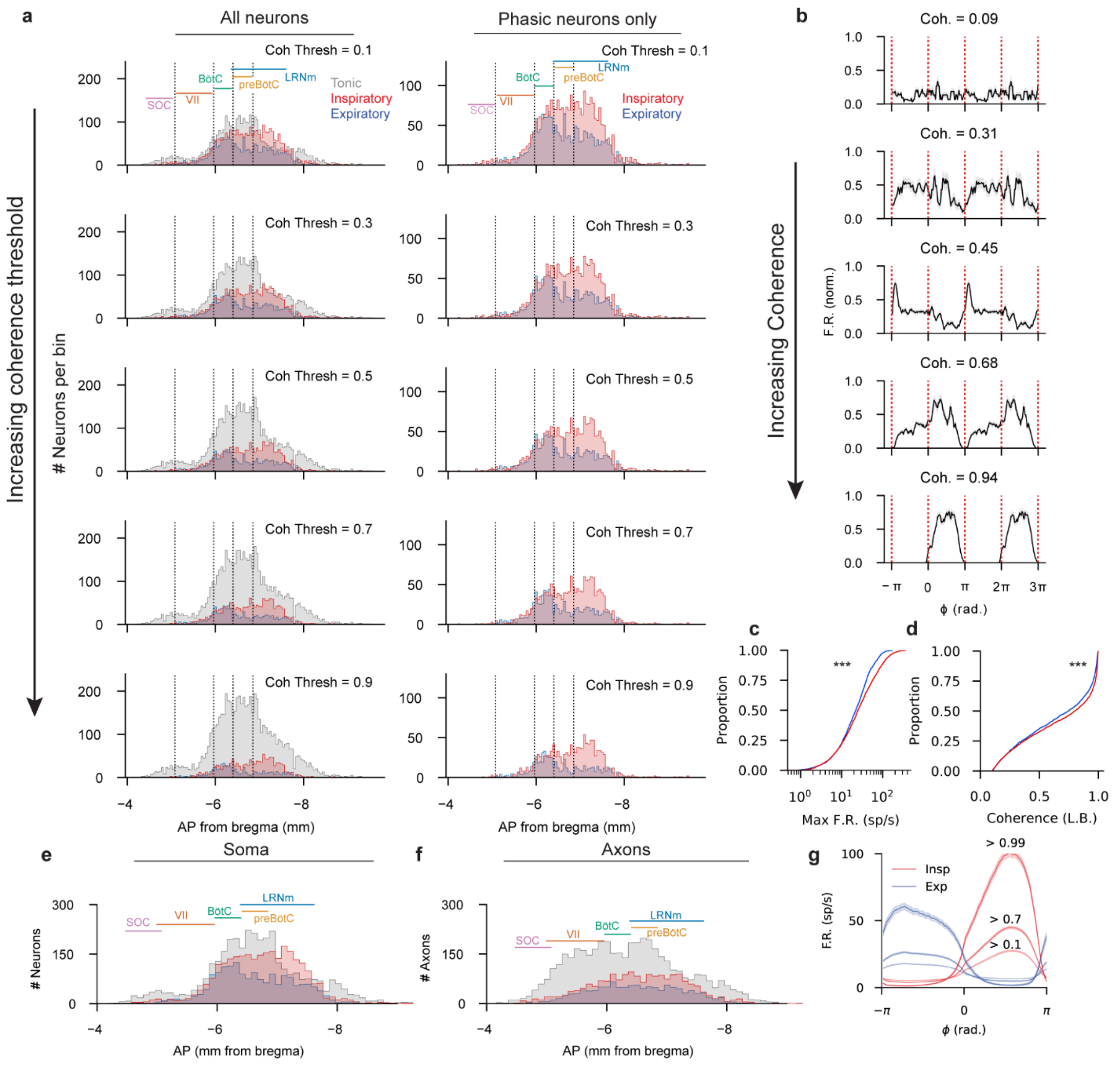

Anatomical distributions are robust to coherence thresholds and the potential of contamination from non-local units. (**a**) Detailed anterior-posterior (AP) distributions of recorded somatic units for varying coherence thresholds. (Left) All recorded units are classified as tonic (grey) inspiratory (red) or expiratory (blue) for increasing coherence thresholds (0.1, 0.3, 0.5, 0.7, 0.9). Units with a lower bound of coherence (C_{lb}) less than the threshold value (i.e. weakly coherent units) are

classified as tonic. Phasic somatic units (i.e., units with a C_{lb} > threshold) were further classified as expiratory or inspiratory based on the phase lag of that unit relative to respiration. (Right) same as (left) but tonic neurons are omitted. Bins are 50μm. AP extent of landmark regions in the ventral medulla are shown, but histograms include units from all regions that overlap at a given AP location (e.g. intermediate reticular nucleus). There is a smooth anterior-posterior gradient in which expiratory neurons are found anteriorly and inspiratory neurons posteriorly. (**b**) Example phase aligned single unit firing rates at varying coherence values. Firing rates are normalized between 0 (no firing) and 1 (maximal firing rate) before taking the mean +/- S.E.M. Two cycles are shown. Inspiration onset (diaphragm activation) is $\phi = 0,2\pi$; inspiration offset/expiration onset (diaphragm cessation) is $\phi = \pi, 3\pi$. (**c**) Maximum firing rate and (**d**) C_{lb} cumulative distributions for all inspiratory (red, n=2,586) expiratory (blue n=1,718) somatic units. (Two-sided Mann-Whitney U test, ***(c) p=4.3e-9, ***(d) p=2.97e-5) Inspiratory units have higher maximum firing rates than expiratory units. (**e**) AP distributions of somatic and (**f**) axonal units, tonic units are defined with C_{lb} threshold <0.1. Binsize is $150\mu m$. Large densities of tonic axons are present rostral near the facial nucleus, and the increased density of expiratory units in the Botzinger region is not seen in axonal units. (**g**) Phase-averaged firing rates of all inspiratory (red) and expiratory (blue) units at three coherence thresholds (0.1,0.7,0.99).

Supplementary Figure 4

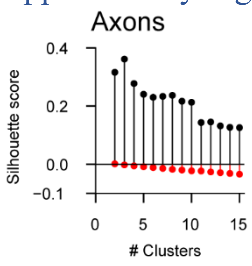

Maximal silhouette score for hierarchical clustering for 2 through 15 computed clusters performed on putatively axonal units. The maximal silhouette score occurs when the number of clusters is 3. Red points indicate shuffled control.

Supplementary Figure 5

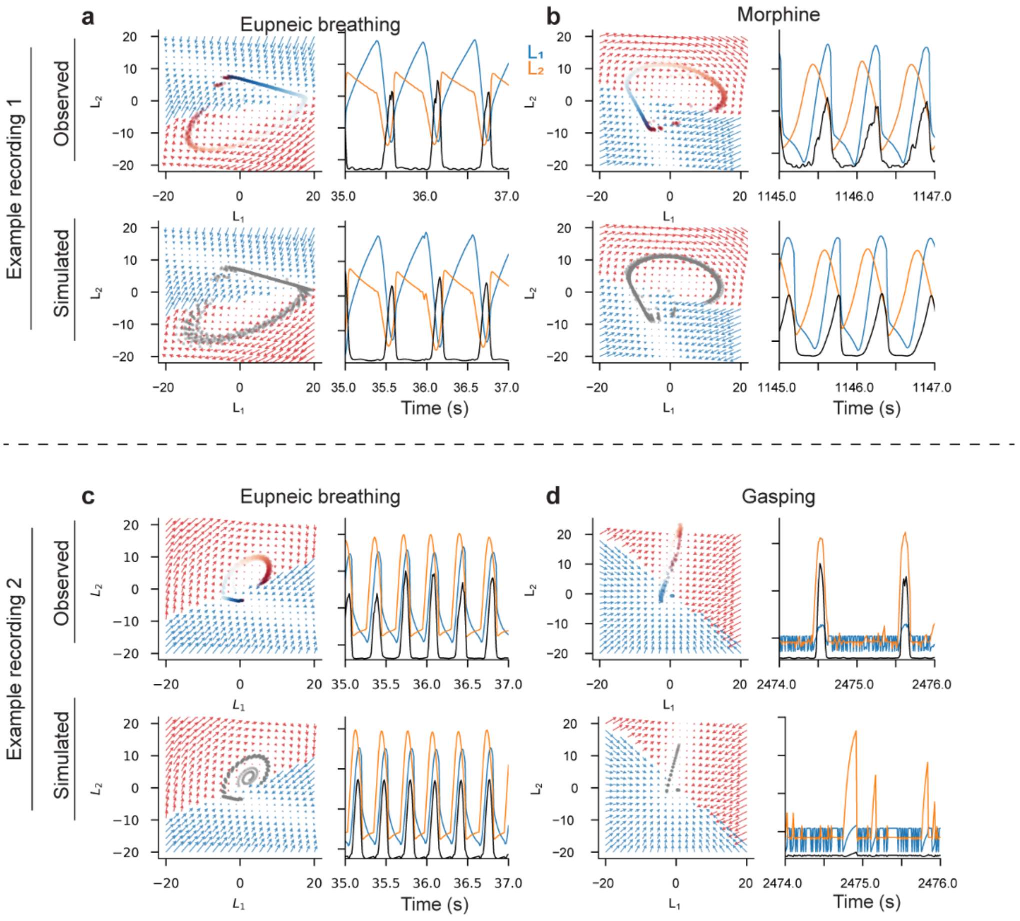

Maintenance of rotational dynamics during morphine administration, and loss of that structure in gasping. (**a**) rSLDS fit and simulation for an example regenerative recording of eupneic breathing. Layout as in Fig. 4e. (**b**) rSLDS fit and simulation for the recording in (a), during morphine. Rotational, regenerative dynamics are maintained during morphine, although the axes of the latent space have been rotated. (**c,d**) as in (a,b), for a different regenerative recording that was subject to hypoxia. Note the loss of rotational dynamics during gasping.

Supplementary Video Legends

Supplementary Video 1

3D reconstruction of all probe insertions, colored by mouse genotype. Colors as in Figure 1.

Supplementary Video 2

Anatomical locations of all recorded neurons. The outline of the entire brain, as well as the facial nucleus (VII) and nucleus ambiguus are shown. Gray neurons on the left side are tonic neurons, light purple neurons on the right side are weakly phasic neurons ($0.1 <$ coherence lower bound $<$ 0.9), and dark purple neurons on the right side are strongly phasic neurons(coherence lower bound $>$ 0.9). Note that the strongly phasic neurons are found in a more restricted region of the ventrolateral medulla than either the tonic or weakly phasic neurons and are not often found within VII. Visualization performed with Brainrender[2]

Supplementary Video 3

Neural population activity evolves through constrained trajectories in a low-dimensional space. Integrated diaphragmatic activity (top) and (middle) activity of the 249 simultaneously recorded neurons in Fig 2. evolve over time. Firing rates are binned at 5ms and smoothed with a Gaussian kernel of s.d. = 15ms. Firing rates are normalized to maximum firing rate for visualization. Purple is 0 firing rate, yellow is maximal. Trajectories evolve over time through constrained PC space (bottom). Color of moving trace in bottom indicates diaphragmatic activity (black is inactive, yellow is maximally active). Video speeds from 15% real time to 50% real time after several seconds of playback. The inspiration off attractor described in Fig. 2 is appreciated in the temporal evolution of the neural population trajectories. A sigh and associated post-sigh eupnea is observed early in the video; the neural population trajectory exhibits an excursion from the typical eupnea trajectory.

Supplementary Video 4

Regions of a stable neural manifold correlates to multiple respiratory features. Each quadrant of the video shows the position of the neural population in the space defined by the leading 3 PCs for a 1000s time period, with each dot representing a single 5ms time bin. Quadrants are colored by the (top left) diaphragmatic activity, (top right) trajectory speed, (bottom left) 4th PC, and (bottom right) respiratory phase.

Supplementary Video 5

Morphine slows neural population trajectories through PC space. Red line shows trajectory evolution at 30% real time speed in control (left) and morphine (right). Colored dots show 50s of overlapping neural states, where each dot is a 5ms bin. Dots are colored by trajectory speed.

Supplementary Video 6

Transition from eupnea to gasping and recovery. Video laid out as in Supplementary Video 3. 97 neurons are shown in the raster. Video plays at 100% real time of recording. Rotational trajectories through the PC space are observed during eupnea. These rotations become ballistic efforts during gasping (brown traces). During recovery, the trajectories (blue) smoothly transition back to normal eupneic rotations.

Supplementary Tables

Supplementary Table 1

Kilosort 3 parameters used for spike sorting.

Supplementary References

1. Sibille, J. et al. High-density electrode recordings reveal strong and specific connections between retinal ganglion cells and midbrain neurons. Nature Communications 13, 5218 (2022).

2. Claudi, F. et al. Visualizing anatomically registered data with brainrender. Elife 10, e65751 (2021).
