# Supplementary material for: Latent neural population dynamics underlying breathing, opioid-induced respiratory depression and gasping
Source: Nat Neurosci. 2024 Jan 5;27(2):259–71. doi: 10.1038/s41593-023-01520-3 (PMC10849970; doi:10.1038/s41593-023-01520-3)
Supplement: Supplementary file 2 — Reporting Summary [file 41593_2023_1520_MOESM2_ESM.pdf]

nature portfolio

Corresponding author(s): Jan-Marino Ramirez

Last updated by author(s): Aug 7, 2023

Reporting Summary

Nature Portfolio wishes to improve the reproducibility of the work that we publish. This form provides structure for consistency and transparency in reporting. For further information on Nature Portfolio policies, see our Editorial Policies and the Editorial Policy Checklist.

Statistics

For all statistical analyses, confirm that the following items are present in the figure legend, table legend, main text, or Methods section.

n/a	Confirmed	
☐	☒	The exact sample size (*n*) for each experimental group/condition, given as a discrete number and unit of measurement
☒	☐	A statement on whether measurements were taken from distinct samples or whether the same sample was measured repeatedly
☐	☒	The statistical test(s) used AND whether they are one- or two-sided<br>*Only common tests should be described solely by name; describe more complex techniques in the Methods section.*
☐	☒	A description of all covariates tested
☐	☒	A description of any assumptions or corrections, such as tests of normality and adjustment for multiple comparisons
☐	☒	A full description of the statistical parameters including central tendency (e.g. means) or other basic estimates (e.g. regression coefficient) AND variation (e.g. standard deviation) or associated estimates of uncertainty (e.g. confidence intervals)
☐	☒	For null hypothesis testing, the test statistic (e.g. *F*, *t*, *r*) with confidence intervals, effect sizes, degrees of freedom and *P* value noted<br>*Give P values as exact values whenever suitable.*
☒	☐	For Bayesian analysis, information on the choice of priors and Markov chain Monte Carlo settings
☒	☐	For hierarchical and complex designs, identification of the appropriate level for tests and full reporting of outcomes
☒	☐	Estimates of effect sizes (e.g. Cohen's *d*, Pearson's *r*), indicating how they were calculated

Our web collection on statistics for biologists contains articles on many of the points above.

Software and code

Policy information about availability of computer code

Data collection	Electrophysiology data was collected with SpikeGLX versions 20201012 through 20221012. Histology data was collected with Olympus VS-ASW version 2.9 software.
Data analysis	Custom python 3 and Matlab 2019 code was used to analyse all data. CatGT versions 1.4.1 through version 3.9 were used to postprocess the electrophysiology data. Kilosort version 3 was used to sort the electrophysiology data. Chronux version 2.12 was used to compute coherences. Open-source packages for these platforms are used throughout, and are indicated or cited in the manuscript. All analysis code is available upon request.

For manuscripts utilizing custom algorithms or software that are central to the research but not yet described in published literature, software must be made available to editors and reviewers. We strongly encourage code deposition in a community repository (e.g. GitHub). See the Nature Portfolio guidelines for submitting code & software for further information.

Data

Policy information about availability of data

All manuscripts must include a data availability statement. This statement should provide the following information, where applicable:

- Accession codes, unique identifiers, or web links for publicly available datasets
- A description of any restrictions on data availability
- For clinical datasets or third party data, please ensure that the statement adheres to our policy

Summary source data are provided in the manuscript. All other data are available upon reasonable request.

Research involving human participants, their data, or biological material

Policy information about studies with human participants or human data. See also policy information about sex, gender (identity/presentation), and sexual orientation and race, ethnicity and racism.

Reporting on sex and gender	N/A
Reporting on race, ethnicity, or other socially relevant groupings	N/A
Population characteristics	N/A
Recruitment	N/A
Ethics oversight	N/A

Note that full information on the approval of the study protocol must also be provided in the manuscript.

Field-specific reporting

Please select the one below that is the best fit for your research. If you are not sure, read the appropriate sections before making your selection.

☒ Life sciences ☐ Behavioural & social sciences ☐ Ecological, evolutionary & environmental sciences

For a reference copy of the document with all sections, see nature.com/documents/nr-reporting-summary-flat.pdf

Life sciences study design

All studies must disclose on these points even when the disclosure is negative.

Sample size	Sample size was not determined a-priori but were chosen to be commensurate with similar studies.
Data exclusions	We excluded experiments in which animals did not survive to the conclusion of the recording paradigm due to respiratory failure, notably in response to hypoxia challenge. We excluded recordings in which large movements of the Neuropixel probe (evident during recording quality control) were detected. Data omitted from particular analyses are noted and explained in the manuscript
Replication	Recordings were repeated across multiple mice (n=36 mice), with multiple recordings per mouse (average 3.8 recordings per mouse). Similar results were observed across recordings.
Randomization	Mice were recorded from randomly based on availability on a given day of recording.
Blinding	Blinding is not applicable as there is no user based scoring of outcomes.

Reporting for specific materials, systems and methods

We require information from authors about some types of materials, experimental systems and methods used in many studies. Here, indicate whether each material, system or method listed is relevant to your study. If you are not sure if a list item applies to your research, read the appropriate section before selecting a response.

Materials & experimental systems

n/a	Involved in the study
☒	☐ Antibodies
☒	☐ Eukaryotic cell lines
☒	☐ Palaeontology and archaeology
☐	☒ Animals and other organisms
☒	☐ Clinical data
☒	☐ Dual use research of concern
☒	☐ Plants

Methods

n/a	Involved in the study
☒	☐ ChIP-seq
☒	☐ Flow cytometry
☒	☐ MRI-based neuroimaging

Animals and other research organisms

Policy information about studies involving animals; ARRIVE guidelines recommended for reporting animal research, and Sex and Gender in Research

Laboratory animals	Male and female mice were used. Jax identification numbers of all animals are given in the main text. Mice aged p46-p155 were used. Animals were housed on a 12/12 light dark cycle with 50% humidity and temperature set to 73 degrees Fahrenheit .
Wild animals	No wild animals were used.
Reporting on sex	Animals of both sexes are used, and data from both sexes is grouped throughout. We do not stratify analyses based on sex as the majority of analyses are performed within recordings, rather than across animals.
Field-collected samples	No field collected samples were used.
Ethics oversight	All procedures were approved by the Seattle Children's Research Institute Institutional Animal Care and Use Committee.

Note that full information on the approval of the study protocol must also be provided in the manuscript.
